# Dereplication of Bioactive Spirostane Saponins from *Agave macroacantha*

**DOI:** 10.1021/acs.jnatprod.1c00663

**Published:** 2021-10-21

**Authors:** Alexandra G. Durán, Odeta Celaj, Francisco A. Macías, Ana M. Simonet

**Affiliations:** † Allelopathy Group, Department of Organic Chemistry, Institute of Biomolecules (INBIO), Campus de Excelencia Internacional (ceiA3), School of Science, University of Cadiz, C/República Saharaui, 7, 11510 Puerto Real, Cadiz, Spain; ‡ Dipartimento di Scienze e Tecnologie Ambientali Biologiche e Farmaceutiche − DiSTABiF, Universitá degli Studi della Campania “Luigi Vanvitelli”, Via Vivaldi 43, 81100 Caserta, Italy

## Abstract

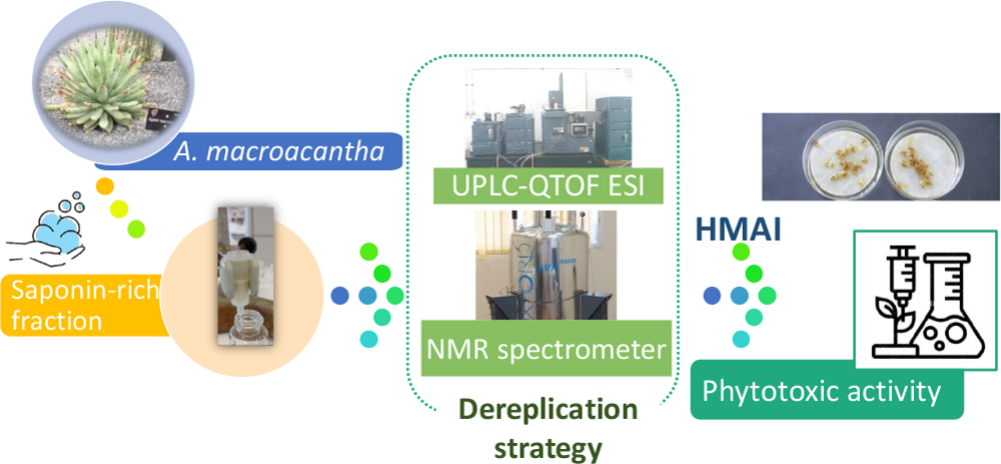

A dereplication strategy using UPLC-QTOF/MS^E^, the HMAI method, and NMR spectroscopy led to the identification of five main steroidal saponins (**1**–**5**), including three previously unknown compounds named macroacanthosides A–C (**3**–**5**), in a bioactive fraction of *Agave macroacantha*. The major saponins were isolated, and some of them together with the saponin-rich fraction were then evaluated for phytotoxicity on a standard target species, *Lactuca sativa*. The inhibition values exhibited by the pure compounds were confirmed to be in agreement with the phytotoxicity of the saponin-rich fraction, which suggests that the saponin fraction could be applied successfully as an agrochemical without undergoing any further costly and/or time-consuming purification processes. The NMR data of the pure compounds as well as of those corresponding to the same compounds in the fraction were comparable, which indicated that the main saponins could be identified by means of this replication workflow and that no standards are required.

In an effort to search for new phytotoxic natural products, steroidal saponins have been investigated recently with some promising results. Hence, the inhibition of root development at high doses, on some occasions, may result in growth stimulation when applied in smaller amounts (hormesis).^1−3^

Steroidal saponins are formed from a hydrophobic sterol skeleton (aglycone) and a hydrophilic carbohydrate chain linked via a glycosidic bond.^4,5^ They usually appear with structurally complex related forms and similar polarity in crude extracts, and their isolation through traditional phytochemical methods remains a considerable challenge. Thus, their prospective commercial use seems to be more suitable when in the form of extracts or enriched fractions. For instance, *Yucca schidigera* extract may be used in some agricultural applications such as livestock nutrition or as an enhancer of yield and quality for certain horticultural crops.^6,7^ It is, however, of the utmost importance to unveil the composition and active principles content in such extracts.

Over the past few years, the separation and characterization of new bioactive natural products, especially when they present similar structures or are found in complex mixtures, has remained a real challenge. Conventional isolation techniques often require large amounts of the starting material as well as time-consuming and costly purification procedures.^8^ In order to overcome such difficulties, several dereplication approaches have been applied to the chemical profiling of natural products. Such procedures are intended to accelerate the identification of the biologically active substances in the extracts and involve clearly defined step-by-step operations or workflows based on their different separation, analytical, and elucidation steps.^9^

Generally, saponin mixtures have been dereplicated by applying chromatographic techniques coupled with mass spectrometry (e.g., UPLC-MS).^10^ Even though this is a highly sensitive and specific technique that allows the identification of compounds without any further isolation, it also presents some significant drawbacks. For instance, for some of the compounds, structural information, such as geometrical isomers, stereoisomers, or the linkage position of sugar moieties, does not allow a fully satisfactory differentiation.^11^ For this reason, HPLC-MS techniques are required to be complemented by NMR spectroscopy to attain an unambiguous and precise determination of a given compound structure. In fact, NMR spectroscopy may be combined with other techniques to achieve the complete elucidation of the compounds of interest. This has enabled NMR-based metabolic profiling studies to increase in their potential use.^12^ Thus, Xiao et al. identified and quantified different lignans in a lignan-rich fraction from *Sambucus williamsii* (a traditional Chinese medicinal plant) using HSQC NMR.^13^ In another research study conducted by García-Pérez et al. different workflows were designed to successfully identify known and unknown metabolites in some complex biological samples employing statistical spectroscopic tools, two-dimensional NMR spectroscopic analysis, multiple hyphenated analytical platforms, and data extraction from existing spectral databases.^14^ Moreover, an identification method for the most representative aglycones in *Agave* species using their ^1^H NMR and heteronuclear multiple bond correlation (HMBC) spectra has been developed, namely, the HMBC method for aglycone identification (HMAI).^4^

Species from the genus *Agave* are known to be natural sources of steroidal saponins. This genus belongs to the family Agavaceae and comprises more than 400 species widely distributed in tropical and subtropical regions throughout the world. Several commercially available specimens of different species from this genus were provided by Desert City Company (Madrid, Spain) and used to produce saponin-enriched fractions that were used for the present work. These extracts have been tested by our research group through wheat etiolated coleoptile bioassays.^3^ As a result, an extract from *Agave macroacantha* Zucc. was found to exhibit superior growth inhibitory activity.

*A. macroacantha* (also known as black-spined agave) is a semelparous plant native to the Tehuacán-Cuicatlán tropical desert of Mexico. It is characterized by succulent leaves, which are arranged in basal rosettes, and by its successful reproductive method, which relies on nocturnal pollinators.^15−17^ Only a small number of studies have been conducted on the isolation and characterization of the steroidal sapogenins and saponins that occur in this species.^18,19^

Given the promising results obtained from the preliminary studies conducted, a dereplication strategy was designed consisting of applying mass spectrometry (UPLC-MS), the HMAI method, and monodimensional NMR experiments to the identification of steroidal saponins from a bioactive fraction of *A. macroacantha*. The major compounds (**1**–**5**) were isolated and their phytotoxicities were evaluated.

## Results and Discussion

Initially, a saponin-enriched fraction from *A. macroacantha* was obtained by solid phase extraction (SPE) using a Strata-X 33 μm polymeric reversed-phase cartridge applied to the organic phase of an initial extraction from the dried plant material obtained using a biphasic solvent (water–*n*-butanol). This enriched fraction was tested in an etiolated wheat coleoptile bioassay,^3^ which allows evaluation of the growth inhibition or stimulation of the undifferentiated plant tissues and results in a strong inhibitory activity (IC_50_ = 32.7 ppm). In previous reports, this effect has been associated with the phytotoxic activity on *Lactuca sativa* of the spirostane saponins present in species of the family Agavaceae.^2,3^ The phytotoxicity studies that have been performed on this type of compound indicated that their observed activities could be defined by the oxygenation of the aglycone backbone, especially at C-12, and also by a sugar chain formed by four or more monosaccharide units. On this basis, the application of enriched fractions containing bioactive saponins instead of using pure compounds seems to be a viable alternative. For this purpose, it would be essential to discern accurately the chemical composition of the active fractions. Toward this goal, the dereplication of the enriched fractions through an exhaustive study of their 1D and 2D NMR spectra using an HMAI method for the aglycones in *A. macroacantha* followed by UPLC-MS analysis was investigated. Furthermore, this study intended to demonstrate that the methodology used would allow the elucidation of the main saponins present in chromatographic fractions of this plant with no additional purification required.

The analysis of the molecular ions and fragmentations obtained through UPLC-QTOF/MS^E^ (Table 1) allowed a number of common features associated with spirostane saponins to be confirmed. The best chromatograms were obtained in the negative ion mode, with formate adducts of the precursor ion detected. The molecular ions [M – H]^−^ and the fragment ions derived from the sequential loss of sugars moieties also were observed. The fragmentation patterns displayed of the sugar residues were in agreement with the presence of a hexose (162 amu), deoxyhexose (146 amu), and/or pentose residue (132 amu). In most cases, the last and most intense fragment corresponds to the aglycone together with the monosaccharide that is directly linked to it. This sugar is usually a hexose residue from the monodesmosidic saponins that are found in *Agave*,^20^ representing the fragment [aglycone – H + 162]^−^.

**Table 1:** UPLC-QTOF/MS^E^ Data Corresponding to a Saponin-Enriched Fraction of *A. macroacantha*

retention time (min)	relative abundance^a^	[M – H]^−^	fragmentation (*m*/*z*)	aglycone
1.398	5.09	771.4140		
2.095	6.03	1207.5341	1075.49, 899.42, 767.38, 605.33	A4
2.392	13.94	1209.5521	1077.51, 901.44, 769.40, 607.35	A3
2.993	21.42	1191.5414	1059.50, 883.43, 751.39, 589.34	A2
3.527	35.79	1193.5555	1061.51, 885.45, 753.40, 591.35	A1

a Percent area of trace vs total area of saponin peaks. Only saponins at over 5% concentration are included.

The HMAI method (HMBC method for aglycone identification)^4^ has been proposed recently for the rapid and reliable identification of aglycones in steroidal saponins of *Agave* spp. Using the ^1^H NMR chemical shifts corresponding to the methyl groups of the aglycone core (these signals are clearly visible due to their high intensity) and their HMBC correlations (^13^C NMR chemical shifts at up to a three-bond distance from the methyl groups), the functional groups and structural features around each methyl group can be identified. Two flowcharts proposed as tools for this method (Figures S1 and S2, Supporting Information) through a number of decision steps that are indicated inside diamonds can provide structural information. When this method is applied to a saponin mixture, the results obtained can be combined with the data provided by the UPLC-QTOF/MS^E^ analysis, which allows the assignment and elucidation of the main saponins with no further purification required.

**Figure 1:**
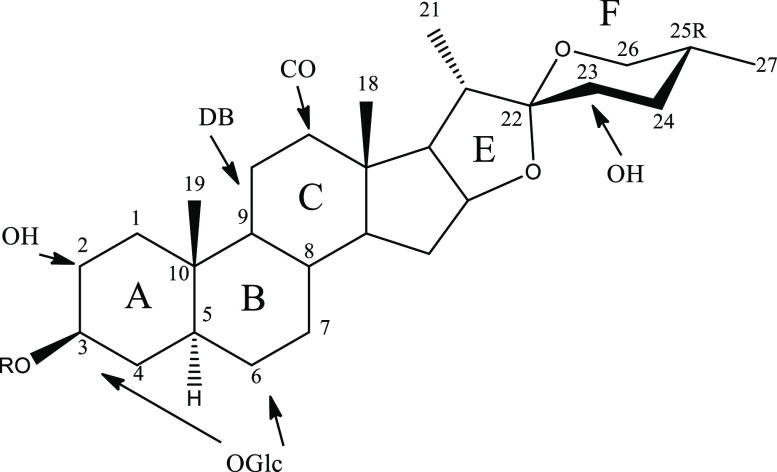
Structural features of *A. macroacantha* saponins as determined by the HMAI method. OH, hydroxy group; DB, double bond; CO, carbonyl group; OGlc, glucopyranosyloxy group.

The ^1^H NMR spectrum of the saponin-rich extract from *A. macroacantha* displayed the characteristic methyl group (singlets and doublets) signals from the aglycone moiety in the upfield region. On the other hand, to enable the identification of minor or overlapped signals, a PS1D spectrum (one-dimensional pure shift) was acquired. This shows the ^1^H NMR signals as singlets and, therefore, displays a clearer image of this area (Figure S3, Supporting Information). Once the correlations of these signals were located in the HMBC spectrum, they were interpreted with the HMAI method using the two flowcharts and the relevant spectroscopic data reported in the literature. The structural features found (Table S1, Supporting Information) showed the presence of spirostane-type saponins with an *R* configuration of C-25, a *trans* fusion of rings A and B (H-5α), either a carbonyl group at the C-12 position or an α,β-unsaturated carbonyl group on this same position, a hydroxy group at C-2 or C-23, and a glucopyranoxyloxy group at C-6 associated with another unit at C-3 (Figure 1).

The HMBC correlations of the most intense doublet (1.32 ppm) and singlet (1.05 ppm), corresponding to the methyl groups at C-21 and C-18, respectively, showed the presence of a carbonyl group at C-12 in the C ring. This functionalization on a spirostane aglycone is in accordance with the main aglycone fragment ion ([aglycone – H + 162]^−^) at *m*/*z* 591.35 (38.6%) obtained through UPLC-QTOF/MS^E^ analysis (Table 1). The HMBC correlations of the doublets at 1.38 ppm (C-21) and at 0.98 ppm (C-18) were in agreement with this same aglycone along with unsaturation between C-9 and C-11 and with the aglycone fragment ion at *m*/*z* 589.34 (24.9%). On the other hand, the fragments at *m*/*z* 607.35 (17.7%) and 605.33 (6.0%) differ from those previously by 16 amu, which led to the proposal of an additional oxygen for these aglycones. HMBC correlations that were observed for the signal at 0.88 ppm were assigned to the C-19 methyl and were in agreement with the functionalization proposed for the *m*/*z* 605.33 fragment ion, involving the hydroxy group with an α configuration at C-2. Moreover, the HMBC correlations of another signal at 0.72 ppm (C-19) was also in an α configuration at C-2. Likewise, the signal relative intensity of this was in agreement with the fragment *m*/*z* 607.35 (17.7%). The presence of a hydroxy group at C-2 caused a slight shielding effect on the methyl groups. Nevertheless, despite the small effect observed on these methyl signals, it allows their assignment for the minor aglycones. Finally, all signals related to methyl groups at C-19 were in agreement with a H-5α configuration.

**Figure 2:**
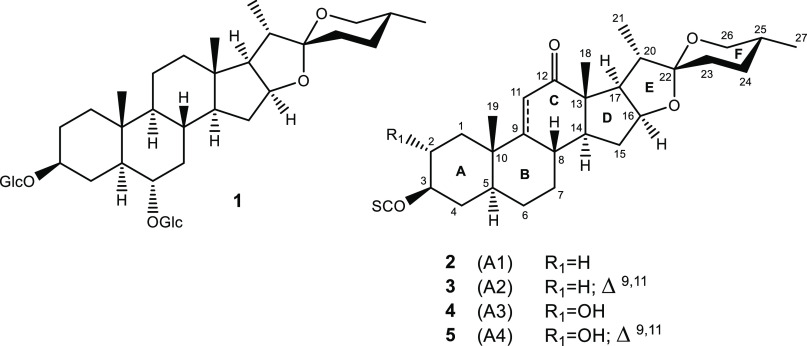
Structures of the main saponins of *A. macroacantha*.

Regarding the F ring, only two signals were assigned to the C-27 methyl group after applying the HMAI method. A major signal (0.66 ppm) was proposed as the overlapping signals from the four saponins in the ^1^H NMR spectrum, since all aglycone functionalizations observed occurred at C-12. The HMBC correlations of these signals allowed confirmation that the F ring corresponded to a 25*R*-spirostane-type saponin. Accordingly, the structures proposed for these four aglycones are as follows: hecogenin (A1), 9-dehydrohecogenin (A2), manogenin (A3), and 9-dehydromanogenin (A4) (Figure 2).

A set of lower intensity correlations in the HMBC spectrum allowed the detection of the ^1^H NMR signals corresponding to the methyl groups in another saponin. The data provided by the HMAI flowchart indicate the presence of a hydroxy group on C-23 from the HMBC correlations of the signal at 0.71 ppm (C-27). On the other hand, the presence of two glucopyranoxyloxy groups on the positions C-23 and C-6 have been also concluded from the signal at 0.59 ppm (C-19). The combination of these structural features led to cantalasaponin-1 (**1**), which has been previously described in *A. macroacantha*(18) and for which mass is consistent with a saponin obtained with a retention time of 1.398 min (5.09%) by UPLC-QTOF/MS^E^ analysis.

Altogether, five saponins exceed 5% relative abundance, which constituted 82.7% of the total saponin-enriched fraction (Table 1). Besides cantalasaponin-1 (**1**), the study of the fragment ions in the rest of the saponins observed by UPLC-QTOF/MS^E^ analysis allowed a sugar moiety, with five monosaccharide units and fragmentation according to three hexose residues (−162 amu), one pentose residue (−132 amu), and one deoxyhexose residue (−146 amu).

This sugar chain also could be observed in the ^1^H NMR spectrum of the saponin-enriched fraction, where the chemical shifts of the anomeric protons appeared in the range of 4.8 to 6.1 ppm. Even though five signals should be observed, some of those signals were obscured, which was explained by the effect of the hydroxy group on the C-2 position of the aglycones A3 and A4 on the closer sugar residues. This deshielding effect of the galactose anomeric proton in the ^1^H NMR spectra has already been described in the literature.^4^

**Figure 3:**
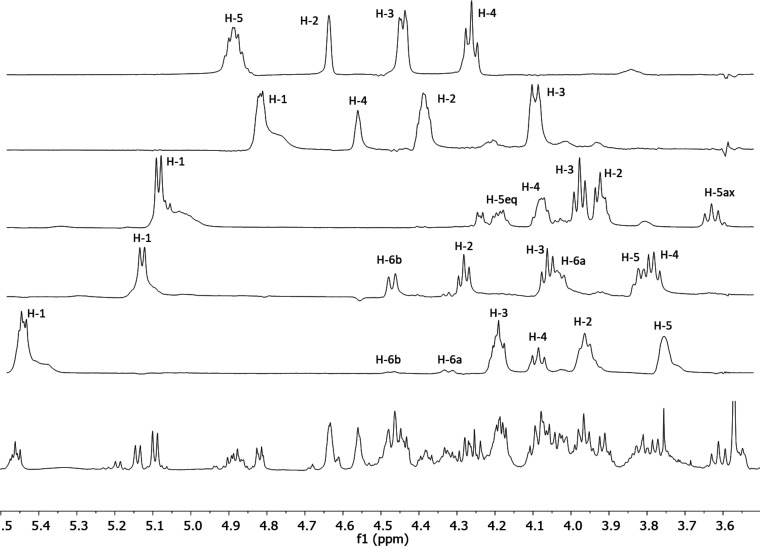
Selected 1D TOCSY NMR spectra (mixing time 100 ms) of the major anomeric signals and the methyl deoxyhexapyranose signal in the saponin-rich fraction of *A. macroacantha*. All the signals displayed were assigned using the 1D TOCSY subspectra corresponding to an acquisition array that included 15, 30, 55, 70, 100, and 150 ms as mixing times.

**Figure 4:**
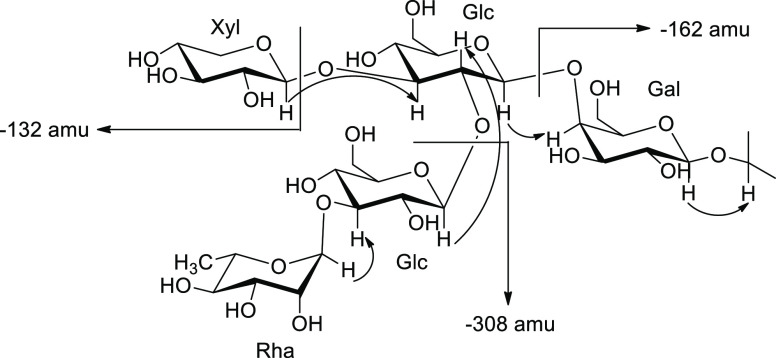
Selected ROE correlations (arrows) of the sugar chain (SC). MS/MS losses were determined according to the MS^E^ analysis.

The five main signals of the ^1^H NMR spectrum with chemical shifts corresponding to the anomeric positions δ 6.07 brs, 5.45 d, 5,14 d, 5,09 d, and 4.82 d were selected to perform the 1D TOCSY experiments.^21^ Thus, the 1D subspectra at different mixing times allowed the assignment of individual spin systems to each sugar moiety (Figure 3). The 1D TOCSY of the broad singlet (6.07 ppm) did not provide any relevant information, because of its small coupling constant. However, the ^1^H NMR spectrum displayed a doublet signal around 1.60 ppm, attributable to the methyl group of a deoxyhexose, and was selected to acquire the 1D TOCSY experiment. The signals that belong to its spin system included the broad singlet at 6.07 ppm. The multiplicity and coupling constants of the signals in the subspectra and the sequences are consistent with two β-glucose, one β-galactose, one β-xylose, and one α-rhamnose moiety. Furthermore, the 1D ROESY spectrum of the aforementioned signals provided correlations between the methine protons with glycosidic bonds. Long-range correlations were observed between H-1_rha_ (δ 6.07) and H-3_glc′_ (δ 4.19), H-1_glc′_ (δ 5.46) and H-2_glc_ (δ 4.29), H-1_glc_ (δ 5.15) and H-4_gal_ (δ 4.57), and H-1_gal_ (δ 4.82) and H-3 of the aglycone (δ 3.83). The sugar chain sequence inferred from the 1D ROESY spectrum was consistent with the fragmentation pattern observed from the UPLC-QTOF/MS^E^ analysis of the main saponins (Figure 4). Thus, the carbohydrate chain was established as α-rhamnopyranosyl-(1→3)-*O*-β-glucopyranosyl-(1→2)-*O*-[β-xylopyranosyl-(1→3)]-*O*-β-glucopyranosyl-(1→4)-*O*-β-galactopyranoside, which had been previously described in other *Agave* spp. including *A. americana*,^22,23^*A. offoyana*,^1^ and *A. brittoniana*.^21,24^

The combination of the previously deduced sugar chain together with the four main aglycones, hecogenin (A1), 9-dehydrohecogenin (A2), manogenin (A3), and 9-dehydromanogenin (A4), led to the determination of four saponins, which showed retention times at 3.527 min (35.79%), 2.993 min (21.42%), 2.392 min (13.94%), and 2.095 min (6.03%) in the UPLC-QTOF/MS^E^ analysis. Hecogenin-3-*O*-{α-l-rhamnopyranosyl-(1→3)-*O*-β-d-glucopyranosyl-(1→2)-*O*-[β-d-xylopyranosyl-(1→3)]-*O*-β-d-glucopyranosyl-(1→4)-*O*-β-d-galactopyranoside} (**2**) has been previously described in *A. americana* as agameroside E.^23^

The remaining three saponins have not been previously reported in the literature. In order to describe their full spectroscopic data, they were subjected to isolation and structural elucidation. A thorough study of the mono- and bidimensional spectra of the pure compounds (Tables 2 and 3) allowed the proposed structures to be confirmed.

**Table 2:** ^13^C and ^1^H NMR Data (*J* in Hz) of the Aglycone Moieties of Compounds **2**–**5** (Pyridine-*d*_5_)^a^

	agameroside E (**2**)		macroacanthoside A (**3**)		macroacanthoside B (**4**)		macroacanthoside C (**5**)	
	δ_C_	δ_H_	δ_C_	δ_H_	δ_C_	δ_H_	δ_C_	δ_H_
1_ax_	36.7	0.69 ddd (13.4, 13.4, 3.8)	35.0	1.18^b^	45.1	1.11^b^	43.6	1.58^b^
1_eq_		1.29 m		1.49 ddd (13.3, 3.7, 3.7)		2.04 dd (12.8, 4.9)		2.24 dd (12.7, 4.9)
2_ax_	29.7	1.53^b^	29.6	1.64^b^	70.2	3.92^b^	69.8	4.03^b^
2_eq_		1.98 brd (12.9)		2.10 brd (12.4)				
3	77.1	3.85 m	76.7	3.84 m	83.9	3.84 m	83.5	3.84 dddd (11.4, 9, 2, 5.0, 5.0)
4_ax_	34.7	1.31 m	34.6	1.36^b^	33.9	1.46 ddd (12.1, 12.1, 12.1)	33.8	1.51^b^
4_eq_		1.77 brd (13.6)		1.83 brd (13.5)		1.85 ddd (13.1, 3.8, 3.0)		1.93 ddd (13.1, 4.3, 3.3)
5	44.5	0.82^b^	42.5	1.05 dddd (12.6, 12.6, 3.0, 3.0)	44.4	0.94^b^	42.5	1.16^b^
6_ax_	28.7	1.10 m (2H)	27.9	1.26^b^	27.9	1.05 m	27.3	1.19^b^ (2H)
6_eq_				1.14 m		1.12 m		
7_ax_	31.8	0.74 dddd (11.7, 11.7, 11.7, 5.2)	32.7	0.89 m	31.6	0.72 m	32.6	0.89 m
7_eq_		1.52^b^		1.71^b^		1.50^b^		1.74^b^
8	34.4	1.72 dddd (11.0, 11.0, 11.0, 3.7)	36.9	2.38 m	33.8	1.71^b^	36.2	2.36 m
9	55.5	0.87^b^	171.4		55.4	0.97^b^	170.6	
10	36.3		39.6		37.3		40.6	
11_ax_	38.1	2.35 dd (13.8, 13.8)	120.0	5.77 d (1.9)	38.1	2.40 dd (12.4, 11.9)	120.3	5.96 d (1.3)
11_eq_		2.21 dd (14.3, 4.9)				2.34 dd (14.3, 5.3)		
12	212.8		204.4		212.6		204.3	
13	55.4		51.4		55.4		51.4	
14	55.9	1.34^b^	52.7	1.69^b^	55.7	1.33^b^	52.7	1.68^b^
15_a_	31.5	1.57 m	31.9	1.64^b^	31.5	1.56^b^	31.9	1.63^b^
15_b_		2.08 ddd (12.5, 6.8, 6.8)		2.17 ddd (11.9, 5.1, 5.1)		2.08 ddd (12.4, 7.0, 5.5)		2.17 m
16	79.8	4.46^b^	80.3	4.51^b^	79.8	4.46^b^	80.3	4.50^b^
17	54.4	2.74 dd (8.7, 6.7)	54.6	2.62 dd (8.8, 7.1)	54.3	2.73 dd (8.7, 6.7)	54.6	2.61 dd (8.7, 7.2)
18	16.2	1.06 s	15.3	0.99 s	16.2	1.05 s	15.3	0.98 s
19	11.8	0.65 s	18.4	0.81 s	13.0	0.73 s	19.5	0.90 s
20	42.7	1.90 dq. (6.9, 6.9)	43.0	1.99 dq (6.9, 6.9)	42.7	1.89 dq. (6.9, 6.9)	43.0	1.98 dq (7.2, 7.2)
21	14.0	1.34 d (6.9)	13.9	1.39 d (6.9)	14.0	1.33 d (6.9)	13.9	1.38 d (6.9)
22	109.4		109.5		109.4		109.5	
23_ax_	31.9	1.60^b^	31.8	1.63^b^	31.9	1.60^b^	31.8	1.62^b^
23_eq_		1.67^b^		1.71^b^		1.66^b^		1.71^b^
24	29.3	1.53 (2H)^b^	29.3	1.54 (2H)^b^	29.3	1.53 (2H)^b^	29.3	1.55 (2H)^b^
25	30.6	1.55^b^	30.6	1.57^b^	30.6	1.55^b^	30.6	1.56^b^
26_ax_	67.0	3.46 dd (10.7, 10.7)	67.0	3.48 dd (10.7, 10.7)	67.0	3.46 dd (10.7, 10.7)	67.1	3.47 dd (10.6, 10.6)
26_eq_		3.57 dd (11.1, 3.8)		3.57 dd (10.7, 4.0)		3.57 dd (11.1, 3.9)		3.58 dd (10.8, 3.6)
27	17.4	0.66 d (5.9)	17.4	0.67 d (5.7)	17.4	0.66 d (5.8)	17.4	0.67 d (5.3)

a The assignments were confirmed by ^1^H–^1^H-COSY, 2D-TOCSY, HSQC, HSQC-TOCSY, and HMBC experiments.

b Overlapped with other signals.

**Table 3:** ^13^C and ^1^H NMR Data (*J* in Hz) of the Sugar Portions of Compounds **2**–**5** (Pyridine-*d*_5_)^a^

	agameroside E (**2**)			macroacanthoside A (**3**)			macroacanthoside B (**4**)			macroacanthoside C (**5**)		
	δ_C_	δ_H (C–H)_	δ_H (O–H)_	δ_C_	δ_H (C–H)_	δ_H (O–H)_	δ_C_	δ_H (C–H)_	δ_H (O–H)_	δ_C_	δ_H (C–H)_	δ_H (O–H)_
		β-d-Gal			β-d-Gal			β-d-Gal			β-d-Gal	
1	102.5	4.83 d (7.7)		102.6	4.83 d (7.6)		103.2	4.88 d (7.9)		103.2	4.89 d (7.6)	
2	73.2	4.40 dd (8.8, 7.8)	6.95	73.2	4.41 dd (8.6, 8.6)	6.97	72.5	4.50 dd (9.0, 7.9)		72.7	4.50 dd (8.6, 8.0)	
3	75.6	4.10^b^	5.07	75.6	4.10^b^	5.08	75.6	4.12^b^	5.30	75.6	4.13 dd (9.6, 3.0)	5.32
4	79.8	4.57 brd (3.9)		79.8	4.58 brd (3.6)		79.0	4.58 brd (3.4)		79.0	4.58 brd (3.3)	
5	75.5	3.99^b^		75.5	3.99^b^		75.9	4.02^b^		75.9	4.02^b^	
6	60.8	4.20 dd (11.2, 5.8)	6.03	60.8	4.21^b^	6.04	60.8	4.18^b^	6.03	60.8	4.18 dd (12.5. 4.6)	6.06
		4.65^b^			4.64^b^			4.57 brd (8.2)			4.57^b^	
		β-d-Glc			β-d-Glc			β-d-Glc			β-d-Glc	
1	104.9	5.16 d (8.0)		104.8	5.16 d (7.9)		104.3	5.22 d (7.9)		104.3	5.22 d (7.9)	
2	81.1	4.30 dd (8.4, 8.9)		81.0	4.31 dd (8.4, 8.8)		80.8	4.26 dd (8.5, 7.9)		80.8	4.27 dd (8.4, 8.9)	
3	87.2	4.07 dd (8.8, 8.7)		87.2	4.08 dd (8.8, 8.8)		87.3	4.05 dd (8.6, 8.8)		87.3	4.05^b^	
4	70.4	3.80 dd (8.9, 8.9)	5.36	70.4	3.79 dd (9.4, 9.4)	5.36	70.4	3.80 dd (7.9, 7.8)	5.34	70.3	3.81^b^	5.35
5	77.7	3.84^b^		77.7	3.82^b^		77.7	3.81 m		77.7	3.80^b^	
6	63.0	4.04 brd (8.2)	6.74	63.0	4.04^b^	6.74	63.0	4.04^b^	6.65	62.9	4.05^b^	6.65
		4.50^b^			4.49^b^			4.48^b^			4.46^b^	
		β-d-Glc′			β-d-Glc′			β-d-Glc′			β-d-Glc′	
1	104.4	5.48 d (8.1)		104.4	5.49 d (8.0)		104.3	5.49 d (8.1)		104.3	5.49 d (8.1)	
2	76.6	3.99 dd (9.0, 9.0)	6.71	76.6	3.98 dd (8.8, 8.8)	6.72	76.5	3.95 dd (8.4, 8.4)	6.77	76.5	3.94 dd (8.7, 8.1)	6.77
3	83.2	4.21 dd (9.1, 9.1)		83.2	4.22 dd (9.1, 9.3)		83.5	4.21 dd (9.1, 9.1)		83.7	4.22 dd (9.2, 8.6)	
4	69.2	4.11 dd (8.8, 8.8)	6.86	69.2	4.10^b^	6.87	69.5	3.96 dd (9.3, 9.3)	6.86	69.5	3.97^b^	6.87
5	78.5	3.76 ddd (9.7, 3.8, 1.6)		78.5	3.77 ddd (9.5, 4.3, 2.4)		78.1	3.72 ddd (9.7, 5.0, 2.4)		78.1	3.71 ddd (9.3, 4.7, 2.2)	
6	62.3	4.33^b^	5.90	62.3	4.35	5.92	62.5	4.36 dd (11.7, 3.4)	5.62	62.5	4.35 dd^b^	
		4.50 da (11.7)			4.50			4.47^b^			4.47^b^	
		β-d-Xyl			β-d-Xyl			β-d-Xyl			β-d-Xyl	
1	105.0	5.11 d (7.7)		105.0	5.12 d (7.7)		105.0	5.12 d (7.7)		105.0	5.12 d (7.7)	
2	75.3	3.94 dd (8.8, 8.8)	8.38	75.3	3.93 dd (9.0, 9.0)	8.38	75.4	3.93 dd (8.3, 8.3)	8.30	75.4	3.93 dd (8.3, 8.3)	8.31
3	78.6	3.99 dd (9.0, 9.0)		78.6	3.99 dd (8.8, 8.8)		78.6	3.98 dd (8.9, 8.9)		78.6	3.98 dd (8.9, 8.9)	7.68
4	70.7	4.10^b^		70.7	4.08^b^		70.7	4.09 ddd^b^		70.7	4.09^b^	
5	67.3	3.63 dd (10.8; 10.8)		67.3	3.63 dd (10.8; 10.8)		67.3	3.61 dd (10.9; 10.9)		67.3	3.61 dd (10.9; 10.9)	
		4.21 dd (11.1; 5.1)			4.20 dd (11.2; 5.5)			4.19 dd (11.2; 5.3)			4.19 dd (11.2; 5.5)	
		α-l-Rha			α-l-Rha			α-l-Rha			α-l-Rha	
1	102.8	6.10 s		102.8	6.10 d (1.6)		102.8	6.10 d (1.5)		102.8	6.10 s	
2	72.4	4.66 d (3.4)		72.4	4.66 brs	6.58	72.5	4.66 dd (3.5, 1.6)		72.5	4.66 brs	6.62
3	72.7	4.47 brd (10.9)		72.7	4.46 dd (9.3, 2.9)	6.26	72.7	4.46 dd (9.3, 3.7)		72.7	4.46^b^	
4	74.2	4.27 dd (9.6, 9.6)		74.2	4.28 dd (9.6, 9.6)		74.2	4.29 dd (9.6, 9.6)		74.2	4.28^b^	6.76
5	69.8	4.91 dq (6.2, 9.7)		69.8	4.92 dq (6.3, 9.3)		69.8	4.90 dq (6.5, 11.0)		69.8	4.89^b^	
6	18.7	1.62 d (6.2)		18.7	1.62 d (6.3)		18.7	1.61 d (6.2)		18.7	1.61 d (6.2)	

a The assignments were confirmed by ^1^H–^1^H-COSY, 2D-TOCSY, HSQC, HSQC-TOCSY, and HMBC experiments.

b Overlapped with other signals.

The chemical shifts of the hydroxy group protons have not been described in the relevant literature for this type of saponins. However, a large number of such signals were detected when assigning the sugar chain spin system from the pure saponins according to the correlations observed in the COSY, TOCSY, and HMBC spectra (Table 3). Deuterated pyridine was used as the solvent for the experiments. This is an aprotic solvent, and when saponins are diluted at low concentrations, the identification of these proton signals in the ^1^H NMR spectrum is possible. Their chemical shifts presented a certain regularity, and the main signals observed belong to the hydroxy groups in the three inner hexoses.

Moreover, compound **2** exhibited identical spectroscopic data and optical activity to those reported in the literature for agameroside E. Therefore, it can be construed that the absolute configurations of the sugar units are d, except for rhamnose, for which the configuration would be l in the three new saponins, named macroacanthosides A–C (Figure 2). These structures were established as 9-dehydrohecogenin-3-*O*-{α-l-rhamnopyranosyl-(1→3)-*O*-β-d-glucopyranosyl-(1→2)-*O*-[β-d-xylopyranosyl-(1→3)]-*O*-β-d-glucopyranosyl-(1→4)-*O*-β-d-galactopyranoside} (**3**), manogenin-3-*O*-{α-l-rhamnopyranosyl-(1→3)-*O*-β-d-glucopyranosyl-(1→2)-*O*-[β-d-xylopyranosyl-(1→3)]-*O*-β-d-glucopyranosyl-(1→4)-*O*-β-d-galactopyranoside} (**4**), and 9-dehydromanogenin-3-*O*-{α-l-rhamnopyranosyl-(1→3)-*O*-β-d-glucopyranosyl-(1→2)-*O*-[β-d-xylopyranosyl-(1→3)]-*O*-β-d-glucopyranosyl-(1→4)-*O*-β-d-galactopyranoside} (**5**).

Thus, it was confirmed that UPLC-QTOF/MS^E^ analysis of the saponin-rich extract from *A. macroacantha* together with the HMAI method for aglycone identification and the selective 1D-TOCSY and ROESY experiments to determine sugar chains have led the structures proposed of the five main saponins in the fraction investigated.

The comparison of the ^1^H and ^13^C NMR spectroscopic data from the pure compounds against those obtained from the HMBC correlations of the enriched fraction (Table S2, Supporting Information) showed that the chemical shifts were almost identical, with an average difference in the mixture with respect to those obtained for the pure products of just 0.01 ppm for ^1^H NMR and 0.1 ppm for ^13^C NMR. Depending on the HMBC resolution, the ^13^C NMR signals with chemical shifts of less than 1 ppm that correlated with the same methyl group may be overlapped, which resulted in a single signal with a chemical shift midpoint. These signals were disregarded when using the HMAI method. The results confirmed the suitability of the method to identify *Agave* aglycones in a mixture.

Concerning the ^1^H and ^13^C NMR spectra signals from the saccharide chain of compounds **2**–**5** (Table 3), a strong influence of a hydroxy group at the C-2 position of the aglycone backbone was observed on the chemical shifts of galactopyranose and the two glucopyranoses. However, the external sugars, xylopyranose, and rhamnopyranose are not affected, and the chemical shifts attributable to these sugar residues were virtually the same for all four compounds. On the other hand, the sugar chain SC1 was not significantly influenced by the presence of a double bond between C-9 and C-11. The ROESY correlations observed in compounds **2** and **4** and their calculated minimum-energy conformations (PCmodel v. 9.2, Bloomington, IN, USA, 2006) were analyzed to explain the influence of the hydroxy group at position C-2.

**Figure 5:**
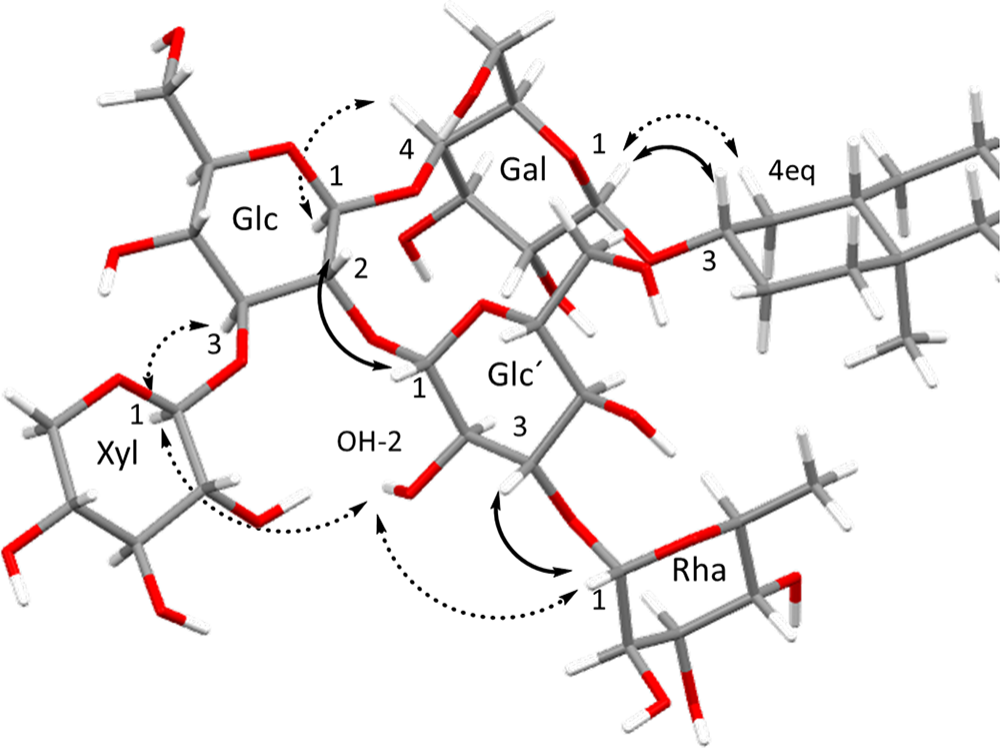
Conformation calculated for compound **2** and NOE effects observed between the different sugar units and the aglycone.

**Figure 6:**
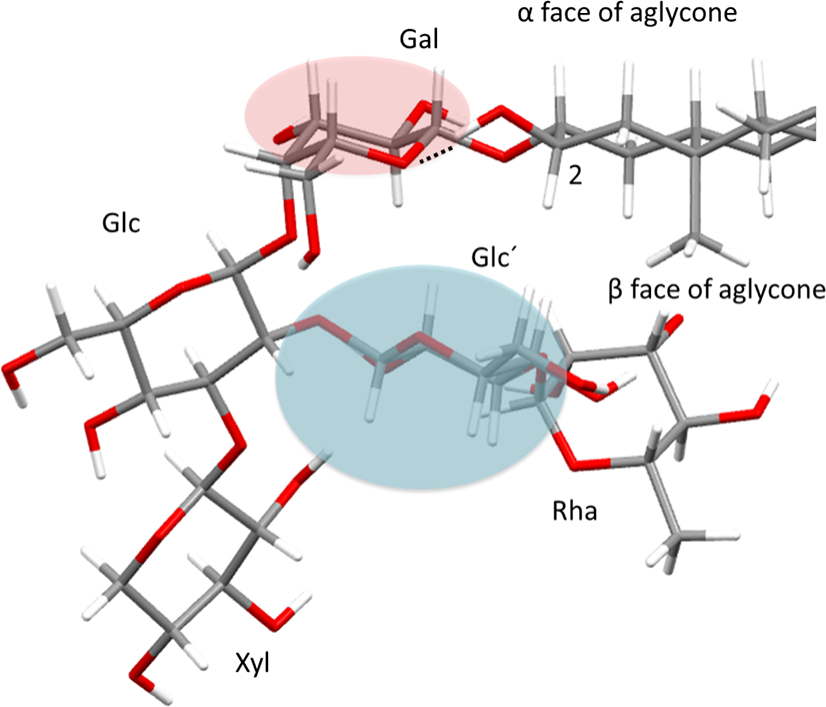
Conformation calculated for compound **4**. The areas highlighted in red and blue colors represent deshielding and shielding of the chemical shifts of the sugar chain compared to compound **2** (which has no hydroxy group at C-2). The dotted line represents the hydrogen bridge between the hydroxy group at C-2 and the galactopyranose oxygen.

The NOE correlations observed in the 1D and 2D ROESY experiments and their intensities indicated the relative spatial arrangement between the two protons. The correlations with the greatest intensities were those corresponding to protons that are closest to the glycosidic bond between sugars (Figure 5), which implies that these will be close in space. Additionally, the NOE correlations observed for the hydroxy group on the C-2 position of the second glucose (Glc′) were crucial. This signal appears as a well-defined doublet at 6.92 ppm (7.9 Hz) for compound **2** and at 6.77 ppm (8.1 Hz) for compound **4**. In the 2D ROESY spectra, both signals displayed correlations with the anomeric protons of xylose and rhamnose, which were observed also in the 1D ROESY spectra of the anomeric signals of these sugars. The conformations proposed for the sugar chain of saponins with and without a hydroxy group on C-2 were similar (Figure 6) and presented spatial arrangements and distances between the nuclei that explained the NOE correlations that have been observed.

The proposed conformation for the sugar chain featured a chain folding that places the rhamnose in the direction of the β-face of the aglycone (Figure 6) and the second glucose unit (Glc′) in a position close to the galactose. Although the conformation of the sugar chain was not altered by the appearance of the hydroxy group on C-2, it is proposed that a variation in the relative position between the aglycone and the chain occurs when the hydroxy group appears at C-2, since the intensity of the NOE effect observed between H-4_eq_ of the aglycone and H-1 of galactose (Figure 5) decreased by about 50%. This intensity drop may be due to the rotation occurring in the glycosidic bond in order to facilitate a hydrogen bridge between the hydroxy group at C-2 and the oxygen of the pyranose in the galactose moiety (Figure 6).

The presence of a hydroxy group at C-2 caused a noticeable deshielding of the chemical shifts of the galactose moiety, while in the second glucose unit (Glc′), it mainly produced a shielding variation (Figure 6). On the other hand, the signals that correspond to the outermost sugar units, i.e., rhamnose and xylose, did not result in significant differences in the ^1^H and ^13^C NMR spectra. This confirmed that the presence of this functional group at the C-2 position of the aglycone skeleton did not modify the spatial arrangement of the saccharide chain when the saponins were examined in pyridine-*d*_5_.

The ^1^H NMR signals of the sugar units in the main saccharide chain of the saponin-rich fraction were determined by 1D TOCSY and 1D ROESY experiments, where the most intense anomeric proton signals, i.e., those corresponding to the chains linked to the aglycones without a hydroxy group on C-2 (**2**, **3**), were selected. Furthermore, it could be verified by comparing the spectroscopic data of the sugar chain in the 2D-TOCSY and ROESY experiments (Table S3, Supporting Information) that the differences between the chemical shifts observed in the ^1^H NMR spectra of the pure compounds **4** and **5** could also be observed in the mixture. On the other hand, the HMBC spectrum of the saponin-rich fraction allowed a large number of signals from the sugar residues to be assigned. The correlations that provided information concerning the glycosidic bonds (the anomeric sugar protons with the glycosided carbons) were more intense than the other correlations (H-1 of the sugars with C-3 and C-5), which represents useful structural information (Figures S4–S6, Supporting Information).

In this way, for instance, a single signal from rhamnopyranose or xylopyranose with intensity 1 could be observed in the region of the anomeric protons. Nevertheless, the signal from the H-1 of the glucopyranose linked to the galactopyranose appeared as two doublets at 5.14 and 5.19 ppm, which corresponded to the saponins containing aglycones without an OH group (**2**, **3**) or with an OH group (**4**, **5**) at C-2 respectively, with a 3:1 ratio between them. This trend also was observed in the MS spectrum of these two types of aglycones.

Moreover, the chemical shifts that belong to the sugar chain in the mixture generally were more shielded than those described in the pure compounds. This variation is analogous to that observed in the ^13^C NMR signals of the aglycone, while the ^1^H NMR reaches a higher value between −0.02 and −0.03 ppm. Such shielding is observed in the signals of the sugar units of all the identified products (**1**–**5**).

The phytotoxicity of the saponin fraction from *A. macroacantha* leaves and that of the pure saponins were evaluated. Due to the limited availability of saponins, only compounds **2**–**4** could be assayed, and lettuce (*L. sativa* L.) was chosen as the model plant to test their phytotoxicities, at 333, 100, 33, 10, 3.3, and 1 μM (Table 4). Since their inhibitory activity on germination or shoot development was not relevant, their effects on root growth were evaluated. Thus, saponins **2** and **3** exhibited more potent inhibitory profiles than the commercial herbicide Logran. Compound **2**, with a carbonyl group at the C-12 position as a structural feature of the aglycone backbone, showed the lowest IC_50_ value (220.9 μM). It seems that the presence of other functionalities on the aglycone skeleton, such as a double bond between C-9 and C-11 (**3**), or a hydroxy group at C-3 (**4**) reduces its phytotoxic activity. This correlation has been reported previously wherein better inhibitory profiles have been exhibited by those saponins with a carbonyl group at the C-12 position of their aglycones.^3^ Likewise, it has been reported that cantalasaponin-1 (**1**) did not exhibit any significant root growth inhibition on *L. sativa*.^1^

**Table 4:** Phytotoxicity of Compounds **2**–**4** Affecting the Growth of the Roots of *Lactuca sativa*

	IC_50_ (μM)	IC_50_ (ppm)	*R*^2^
agameroside E (**2**)	220.9	263.7	0.9633
macroacanthoside A (**3**)	299.5	357.0	0.9878
macroacanthoside B (**4**)	533.1^a^	645.0	0.9851
saponin-rich extract		384.2	0.9923
Logran	444.3^a^		0.9954

a These data were not adjusted to a dose–response curve.

The dereplication of the saponins in the enriched fraction by combining NMR and MS techniques has allowed us to propose the structure of five main saponins (**1**–**5**) without any further purification requirements. Moreover, three new saponins (**3**–**5**) have been identified. The comparison of the spectroscopic data from the pure compounds against those corresponding to the mixture has allowed corroboration that HMAI is a suitable method to identify aglycones in *Agave* spp. On the other hand, the analysis of the NOE correlations between the sugar moieties and the aglycone to determine their spatial arrangements has confirmed the stability of the three-dimensional structures of the sugar chains regardless of the aglycone functionalization. This has allowed the separate analysis of the spectroscopic data of the sugar chain in the mixture, where only the chemical shifts resulting from the presence of a hydroxy group at the C-2 position of the aglycone were considered relevant. Furthermore, phytotoxicity bioassays showed that the presence of a carbonyl group at the C-12 position of the aglycone backbone is a key feature regarding the resultant bioactivity. Moreover, the activity displayed by the saponin-rich extract was similar to that corresponding to some of the isolated saponins on their own. This confirmed that the saponin-rich fraction could be applied with no further purification being required.

## Experimental Section

### General Experimental Procedures

Optical rotations were measured on a JASCO P-2000 polarimeter using methanol as solvent. The exact masses were measured on a UPLC-QTOF ESI (Waters Xevo G2, Manchester, UK) high-resolution mass spectrometer (HRESI-TOFMS). The 1D and 2D NMR spectra were recorded on an Agilent INOVA-600 spectrometer equipped with a 5 mm ^1^H–^13^C–^15^N cryoprobe. The ^1^H (599.772) and ^13^C (150.826) NMR spectra were recorded in pyridine-*d*_5_ (Merck, Darmstadt, Germany) at room temperature. The chemical shifts are given on the δ scale and are referred to the residual pyridine (δ_H_ 8.70, 7.55, 7.18 and δ_C_ 149.84, 135.60, 123.48).

The acetic acid and *n*-butanol were supplied by Panreac Química S.A. (Castellar del Vallés, Barcelona, Spain). The methanol, *n*-hexane, and chloroform were obtained from VWR International (Radnor, PA, USA). The TLC silica 60 F_254_ and TLC Si gel F_254_S RP-18 plates were purchased from Merck (Darmstadt, Germany) and used to monitor the isolation processes. The compounds were visualized under UV_254/366_ light and after spraying them with H_2_SO_4_–H_2_O–HOAc (4:16:80 v/v/v). For further purification, preparative TLC silica gel 60 F_254_ (0.25 mm) and TLC silica gel RP-18 F_254_S (0.25 mm) were used, and they were also supplied by Merck (Darmstadt, Germany).

### Plant Material

The *A. macroacantha* leaves were authenticated and supplied in November 2017 by Desert City S. L. company (CIF B86691474, Madrid, Spain). GPS coordinates were 40.59897539554237, −3.5823863738311497. A reference sample of powdered plant material and *n*-ButOH extract is available in our laboratory labeled as DC2017-M14.

### Extraction and Isolation

The dried leaves (4.33 g) were moistened in 27 mL of water for 2 h, and then the same amount of *n*-butanol was added. The solution was kept at room temperature for 24 h for their extraction. Then, the same volume of water was added a second time, and the extract was kept under continuous slow stirring for another 24 h. After decanting both phases, the solvent was removed from the *n*-butanol layer under vacuum to yield 375.6 mg (8.7%) of crude extract. Part of the crude extract (92.7 mg) was subjected to SPE using a Strata-X 33 μm polymeric reversed-phase cartridge (Phenomenex) with different aliquots of 30 mg as maximum load quantity each time. This extract was purified using different water:methanol ratios as eluent to obtain the saponin-rich fraction (46.8 mg, 50.5%, 2:8, water–methanol). This fraction was purified using preparative TLC on silica gel 60 F_254_ 0.25 mm plates, using *n*-butanol–HOAc–water (5:1:5) as eluent and 16.0 mg load samples (this process was performed in duplicate). Seven fractions were collected (F1–F7), and those from the two preparative plates with the same TLC patterns were combined; the presence of saponins was confirmed by NMR experiments. Fraction F1 (6.3 mg, 19.7%) led to the isolation of macroacanthoside B (**4**, 2.5 mg) and macroacanthoside C (**5**, 1.5 mg) using preparative TLC on Si gel RP-18 F_254_S plates and water–acetone (4:6) as solvent. Likewise, fraction F2 (9.0 mg, 28.1%) under the same purification conditions allowed the isolation of agameroside E (**2**, 4.0 mg) and macroacanthoside A (**3**, 3.0 mg).

#### Macroacanthoside A (**3**):

[α]^25^_Na_ −26.4 (*c* 0.20, MeOH); ^1^H and ^13^C NMR, see Tables 2 and 3; HRESIMS *m*/*z* 1191.5435 [M – H]^**–**^ (calcd for C_56_H_87_O_27_, 1191.5435); ESIMS (negative ion mode) *m*/*z* 1191 [M – H]^−^; MS^E^*m*/*z* 1059 [M – H – 132]^−^, 883 [M – H – 146 – 162]^−^, 751 [M – H – 132 – 146 – 162]^−^, 589 [M – H – 132 – 146 – 162 × 2]^−^.

#### Macroacanthoside B (**4**):

[α]^25^_Na_ −17.8 (*c* 0.23, MeOH); ^1^H and ^13^C NMR, see Tables 2 and 3; HRESIMS *m*/*z* 1209.5536 [M – H]^**–**^ (calcd for C_56_H_87_O_27_, 1209.5540); ESIMS (negative ion mode), *m*/*z* 1209 [M – H]^−^; MS^E^*m*/*z* 1077 [M – H – 132]^−^, 901 [M – H – 146 – 162]^−^, 769 [M – H – 132 – 146 – 162]^−^, 607 [M – H – 132 – 146 – 162 × 2]^−^.

#### Macroacanthoside C (**5**):

[α]^25^_Na_ −15.2 (*c* 0.085, MeOH); ^1^H and ^13^C NMR, see Tables 2 and 3; HRESIMS *m*/*z* 1207.5369 [M – H]^**–**^ (calcd for C_56_H_87_O_28_, 1207.5384); ESIMS (negative ion mode), *m*/*z* 1209 [M – H]^−^; MS^E^*m*/*z* 1075 [M – H – 132]^−^, 899 [M – H – 146 – 162]^−^, 767 [M – H – 132 – 146 – 162]^−^, 605 [M – H – 132 – 146 – 162 × 2]^−^.

### UPLC-QTOF/MS^E^ Analysis

The exact masses of the saponins were measured using a UPLC-QTOF ESI (Waters Xevo G2, Manchester, UK) high-resolution mass spectrometer (HRESI-TOFMS). An ultra-high-performance liquid chromatograph was equipped with an Acquity UPLC HSS T3 1.8 μm, 2.1 × 100 mm column attached to an Acquity UPLC HSS T3 1.8 μm, 2.1 × 5 mm VanGuard precolumn maintained at 45 °C. The mobile phases were water (A) and acetonitrile (B), each containing 0.1% (v/v) formic acid. The elution conditions were as follows: 60% A (0–0.5 min); A from 60% to 50% (0.5–6.0 min); A from 50% to 95% (6.0–7.0 min); 95% A (7.0–7.5 min); A from 95% to 60% (7.5–8.0 min), and maintenance in 60% A (8.0–10.0 min) to condition the column for the next injection. A constant 0.4 mL/min flow was applied. The autosampler temperature was set at 10 °C, and the injection volume was 5 μL.

Electrospray ionization in the negative polarity mode (ESI^–^) was used with the following settings: sample probe capillary voltage 2800 V, sampling cone voltage 30 V; source temperature 120 °C, and desolvation temperature 450 °C. Desolvation and cone gas with flow rates of 850 and 10 L/h, respectively, were used. The data were acquired in the centroid mode using MS^E^ (low collision energy 6 eV, high collision energy ramp 20–80 eV) over a mass range of *m*/*z* 100–2000 and a retention time range of 0–10.0 min with a 0.5 s scan time. The raw data files were processed using MassLynx version 4.1 (Waters Inc., Milford, MA, 2013). The stock solution (1000 ppm) of the saponin-rich fraction was prepared in water–acetonitrile (6:4). All the samples were injected as a 1:15 dilution (66.7 ppm) and filtered through a PTFE syringe filter (0.22 μm) prior to analysis.

### Molecular Modeling Calculations

The PCModel 9.2 application was used to calculate the minimum energy conformers.^25^ The conformers elaborated from the molecular mechanics GMMX calculations were refined, and those with energies higher than 3.5 kcal/mol with respect to the minima were disregarded. The 3D molecular models were constructed from the lowest energy conformers using Mercury 3.5.1 software.

### Etiolated Wheat Coleoptile Bioassay

Seeds of wheat (*Triticum aestivum* L. cv. Catervo) were sown on water-moistened 15 cm diameter Petri dishes and grown away from light at 22 ± 1 °C for 4 days. The assay was carried out according to the methodology previously described in the literature.^3,26^ Coleoptile elongation was measured by the digitalization of the images, and the data were analyzed statistically using Welch’s test. The saponin fraction from *A. macroacantha* was dissolved in DMSO (0.5% v/v), and dilutions were prepared in a phosphate–citrate buffer solution containing 2% sucrose adjusted to 200, 100, 50, 25, and 12.5 ppm. The commercial herbicide Logran, a combination of *N*^2^-*tert*-butyl-*N*^4^-ethyl-6-methylthio-1,3,5-triazine-2,4-diamine (terbutryn, 59.4%) and 1-[2-(2-chloroethoxy)phenylsulfonyl]-3-(4-methoxy-6-methyl-1,3,5-triazin-2-yl)urea (triasulfuron, 0.6%), was used as the positive control sample under the same conditions above-described. A buffered nutritive aqueous solution with DMSO (0.5% v/v) was used as negative control.

### Phytotoxicity Bioassay

The phytotoxicity of both the pure saponins at 333, 100, 33, 10, 3.3, and 1 μM concentrations and the saponin fraction at 400, 200, 100, 50, and 25 ppm was assayed on *L. sativa* L. (lettuce) as reported in the literature.^2^ As in the coleoptile bioassay, the herbicide Logran was used as the positive control sample, and a buffered nutritive aqueous solution containing 0.5% DMSO and none of the test compounds was used as the negative control sample. The parameters to be evaluated (i.e., germination rate, root and shoot length) were registered and statistically analyzed using Fitomed software.^27^ Welch’s test, with significance set at 0.01 and 0.05, was applied to the data. The IC_50_ values were calculated using the GraphPad Prism v. 5.00 software package (GraphPad, San Diego, CA, USA). The data were then adjusted to a sigmoidal dose–response model (constant slope), where possible, and goodness of fit was described by the regression coefficient (*R*^2^).

## Supporting Information Available

Tables with concluding remarks obtained from NMR data of the saponin-enriched fraction and HMAI method; 1D and 2D NMR spectra of saponin-enriched fraction, flowcharts, and tables of ^13^C chemical shifts used for HMAI; ^1^H and ^13^C NMR spectra of new compounds **3**–**5** (PDF)
