# Supplementary material for: Dereplication of Bioactive Spirostane Saponins from Agave macroacantha
Source: J Nat Prod. 2021 Oct 21;84(11):2904–13. doi: 10.1021/acs.jnatprod.1c00663 (PMC8630797; doi:10.1021/acs.jnatprod.1c00663)
Supplement: Supplementary file 1 — np1c00663_si_001.pdf [file np1c00663_si_001.pdf]

Supporting information

Dereplication of Bioactive Spirostane Saponins from *Agave macroacantha*

Alexandra G. Durán,[†] Odeta Celaj,[‡] Francisco A. Macías,*[,†] and Ana M. Simonet*[, †]

[†]Allelopathy Group, Department of Organic Chemistry, Institute of Biomolecules (INBIO), Campus de Excelencia Internacional (ceiA3), School of Science, University of Cadiz, C/ República Saharaui, 7, 11510 Puerto Real, Cadiz, Spain

[‡]Dipartimento di Scienze e Tecnologie Ambientali Biologiche e Farmaceutiche – DiSTABiF, Universita` degli Studi della Campania “Luigi Vanvitelli”, Via Vivaldi 43, 81100 Caserta, Italy

* E-mail: ana.simonet@uca.es; famacias@uca.es

Table of Contents

Table S1. Correlations between methyl groups and nearby carbons observed in the HMBC spectrum of the saponin fraction of *A. macroacantha* and HMAI interpretation 3

Table S2. Comparison of the selected signals from the ^{1}H and ^{13}C NMR of the pure compounds **1**–**5** with the HMBC data from the extract (pyridine-d_5) 4

Table S3. Comparison of the ^{1}H NMR data (J in Hz) of the sugar moieties in compounds **1**, **2**, and **4** (pyridine-d_5)[a,b] in the saponin-enriched fraction and the pure compounds 5

Table S4. HMAI Table of ^{13}C NMR chemical shifts for doublets 7

Table S5. HMAI Table of ^{13}C NMR chemical shifts for singlets 8

Figure S1. HMAI Flowchart of doublets with notes 9

Figure S2. HMAI Flowchart of singlets with notes 10

Figure S3. ^{1}H NMR and Pure shift 1D NMR spectra of saponin-enriched fraction of *A. macroacantha* (0.55 to 1.40 ppm; 600 MHz, Pyridine-d_5) 11

Figure S4. HMBC of saponin-enriched fraction of *Agave macroacantha* for sugar residues (70 to 100 ppm; 600 MHz, Pyridine-d_5) 12

Figure S5. HMBC of saponin-enriched fraction of *Agave macroacantha* for sugar residues (102.0 to 106.5 ppm; 600 MHz, Pyridine-d_5) 13

Figure S6. HMBC of saponin-enriched fraction of *Agave macroacantha* for sugar residues (59.5 to 68 ppm; 600 MHz, Pyridine-d_5) 14

Figure S7. HMBC of saponin-enriched fraction of *Agave macroacantha* (215 to 100 ppm; 600 MHz, Pyridine-d_5) 15

Figure S8. HMBC of saponin-enriched fraction of *A. macroacantha* (70 to 25 ppm; 600 MHz, Pyridine-d_5) 16

Figure S9. 2D TOCSY spectrum of saponin-enriched fraction of *A. macroacantha* (4.80 to 6.10 ppm; 600 MHz, Pyridine-d_5) 17

Figure S10. Selected 1D ROESY spectra of saponin-enriched fraction of *A. macroacantha* (600 MHz, Pyridine-d_5) 18

Figure S11. 2D ROESY spectrum of saponin-enriched fraction of *A. macroacantha* (4.80 to 6.10 ppm; 600 MHz, Pyridine-d_5) 19

Figure S12. HRESI MS (negative mode) of Macroacanthoside A (**3**) 20

Figure S13. ^{1}H NMR spectrum of Macroacanthoside A (**3**) (600 MHz, Pyridine-d_5) 21

Figure S14. ^{13}C NMR spectrum of Macroacanthoside A (**3**) (600 MHz, Pyridine-d_5) 22

Figure S15. HRESI MS (negative mode) of Macroacanthoside B (**4**) 23

Figure S16. ^{1}H NMR spectrum of Macroacanthoside B (**4**) (600 MHz, Pyridine-d_5) 24

Figure S17. ^{13}C NMR spectrum of Macroacanthoside B (**4**) (600 MHz, Pyridine-d_5) 25

Figure S18. HRESI MS (negative mode) of Macroacanthoside C (**5**) 26

Figure S19. ^{1}H NMR spectrum of Macroacanthoside C (**5**) (600 MHz, Pyridine-d_5) 27

Table S1. Correlations Between Methyl Groups and Nearby Carbons Observed in the HMBC Spectrum of the Saponin Fraction of *A. macroacantha* and HMAI Interpretation.

^{1}H NMR signal	HMBC correlations				Methyl assig.	Flowchart information
Major signals						
1.38 d(D8)	42.9	54.5(D6)	109.5(D5)		C-21	SP C9 DB C12 CO
1.32 d(D8)	42.6	54.3(D6)	109.3(D5)		C-21	SP C12 CO
1.31 d	o	o	o		C-21	SP C12 CO
1.05 s(S2)	54.3	55.6	212.7(S1)		C-18	SP C12 CO
1.04 s	o	o	o		C-18	SP C12 CO
0.98 s	51.3	52.6	54.5	204.3(S3)	C-18	SP C9 DB C12 CO
0.80 s	35.0	39.5	42.5	171.3(S5)	C-19	H-5α; C9 DB C12 CO
0.72 s	37.2	44.7	55.3(S9)		C-19	Check data→ H-5α C2 OHα
0.66 d(D2)	29.2(D1)	30.5	66.9(D1)		C-27	SP C25R
0.64 s	36.4	44.4	55.5(S9)		C-19	Check data→ H-5α
Minor signals						
1.15 d	35.8	62.5	111.7(D9)		C-21	Check data→ SP C23 OHα
0.93 s	40.3	41.3	56.3	62.5(S11)	C-18	Check data→ C6 OGlc α
0.88 s	40.6	42.4	43.5	170.5(S4)	C-19	H-5α; C2 OHα. C9 DB C12 CO
0.71 d	31.8(D1)	38.9(D13)	66.0		C-27	SP C23 OHα
0.59 s	36.7	37.4	50.9(S13)	53.8	C-19	C3 OGlcβ; C6 OGlc α [a]

[a]With a signal in the ^{1}H NMR spectrum at 3.34 ppm; *brd*; 12 Hz (S15).
C#: position with functionalization; SP spirostane, α, β, R: configurations, OH hydroxy group; DB: double bond; CO; carbonyl group; OGlc: glucopyranosyloxy group.
Decisions from the flowchart are indicated inside diamonds.

Table S2. Comparison of the selected signals from the ^{1}H and ^{13}C NMR of the pure compounds[a] **1**–**5** with the HMBC data from the extract[b] (pyridine-d_5).

	**Cantalasaponin-1 (1)**		**Agameroside E (2)**		**Macroacanthoside A (3)**		**Macroacanthoside B (4)**		**Macroacanthoside C (5)**	
	$\delta_{H/C}^{a}$	$\delta_{H/C}^{b}$	$\delta_{H/C}^{a}$	$\delta_{H/C}^{b}$	$\delta_{H/C}^{a}$	$\delta_{H/C}^{b}$	$\delta_{H/C}^{a}$	$\delta_{H/C}^{b}$	$\delta_{H/C}^{a}$	$\delta_{H/C}^{b}$
Me-19	*0.59 s*	*0.58 s*	*0.65 s*	*0.64 s*	*0.81 s*	*0.80 s*	*0.73 s*	*0.72 s*	*0.90 s*	*0.88 s*
1	37.5	37.4	36.7	36.4*	35.0	35.0	45.1	44.7*	43.6	43.5
5	50.9	50.9	44.5	44.4	42.5	42.5	44.4		42.5	42.4
9	53.8	53.8	55.5	55.5	171.4	171.3	55.4	55.3	170.6	170.5
10	36.7	36.7	36.3	36.4*	39.6	39.5	37.3	37.2	40.6	40.6
Me-18	*0.94 s*	*0.93 s*	*1.06 s*	*1.05 s*	*0.99 s*	*0.98 s*	*1.05 s*	*1.04 s*	*0.98 s*	*0.97 s*
12	40.4	40.3	212.8	212.7	204.4	204.3	212.6	o-**2**	204.3	o-**3**
13	41.3	41.3	55.4	55.6*	51.4	51.3	55.4	o-**2***	51.4	o-**3***
14	56.4	56.3	55.9		52.7	52.6	55.7		52.7	
17	62.5	62.5	54.4	54.3	54.6	54.5	54.3	o-**2**	54.6	o-**3**
Me-21	*1.16 d*	*1.15 d*	*1.34 d*	*1.32 d*	*1.39 d*	*1.38 d*	*1.33 d*	*1.31 d*	*1.38 d*	*1.36 d*
17	62.5	62.5	54.4	54.3	54.6	54.5	54.3	o-**2**	54.6	o-**3**
20	35.8	35.8	42.7	42.6	43.0	42.9	42.7	o-**2**	43.0	o-**3**
22	111.7	111.7	109.4	109.3	109.5	109.5	109.4	o-**2**	109.5	o-**3**
Me-27	*0.72 d*	*0.71 d*	*0.66 d*	*0.66 d*	*0.67 d*	*0.66 d*	*0.66 d*	*0.66 d*	*0.67 d*	*0.66 d*
24	38.8	38.9	29.3	29.2	29.3	o-**2**	29.3	o-**2**	29.3	o-**2**
25	31.8	31.8	30.6	30.5	30.6	o-**2**	30.6	o-**2**	30.6	o-**2**
26	66.0	66.0	67.0	66.9	67.0	o-**2**	67.0	o-**2**	67.1	o-**2**

* Overlapping HMBC signals.

Table S3. Comparison of the ^{1}H NMR data (*J* in Hz) of the sugar moieties in compounds **1**, **2** and **4** (pyridine-d_5)[a,b] in the saponin-enriched fraction and the pure compounds.

	Sugar chain without OH-2 aglycone (2)		Sugar chain without OH-2 aglycone in the mix (2)		Sugar chain with OH-2 aglycone (4)		Sugar chain with OH-2 aglycone in the mix (4)	
		β-D-Gal		β-D-Gal		β-D-Gal		β-D-Gal
1	102.5	4.83 d (7.7)	102,5	4.82 d (7.6)	103,2	4.88 d (7.9)	103,2	4.86→83.8 (C-3Aglyc)
2	73.2	4.40 dd (8.8, 7.8)		4.39 dd (7.5, 11.1)	72,5	4.50 dd (9.0, 7.9)		4.47
3	75.6	4.10[b]	75,5	4.08 dd (9.6, 3.3)	75,6	4.12[b]	75,6	4.11
4	79.8	4.57 br d (3.9)	79,7	4.56 br d (4.0)	79,0	4.58 br d (3.4)	79,0	4.57
5	75.5	3.99[b]	75,5	3.98 m	75,9	4.02[b]		4.02*
6	60.8	4.20 dd (11.2, 5.8)<br>4.65[b]	60,7		60,8	4.18[b]<br>4.57 br d (8.2)		
		β-D-Glc		β-D-Glc		β-D-Glc		β-D-Glc
1	104.9	5.16 d (8.0)	104,7	5.14 d (7.8)	104,3	5.22 d (7.9)		5.19→79.0 (C-4Gal)
2	81.1	4.30 dd (8.4, 8.9)	80,9	4.29 dd (8.6, 8.6)	80,8	4.26 dd (8.5, 7.9)		4.24
3	87.2	4.07 dd (8.8, 8.7)	87,1	4.06 dd (8.5, 8.5)	87,3	4.05 dd (8.6, 8.8)		4.03
4	70.4	3.80 dd (8.9, 8.9)		3.77 dd (10.0, 8.8)	70,4	3.80 dd (7.9, 7.8)		3.78
5	77.7	3.84[b]	77,5	3.80[b]	77,7	3.81 m	77,6	
6	63.0	4.04 brd (8.2)<br>4.50[b]	62,9	4.02 brd (11.1)<br>4.47 brd (10.9)	63,0	4.04[b]<br>4.48[b]		
					**Cantalasaponin-1 (1)**		**Cantalasaponin-1 (1) in mix**	
		β-D-Glc´		β-D-Glc´		β-D-Glc		β-D-Glc
1	104.4	5.48 d (8.1)	104,4	5.45 d (8.0)	106.4	4.85 d (7.8)	106.3	4.83→80.0 (C-6Aglyc)
2	76.6	3.99 dd (9.0, 9.0)	76,5	3.96 dd (9.1, 9.1)	75.7	4.01 dd (7.8, 8.3)		Overlapped
3	83.2	4.21 dd (9.1, 9.1)	83,2	4.19 dd (8.8, 8.8)	78.5	4.21[b]	78.4	4.21
4	69.2	4.11 dd (8.8, 8.8)		4.09 dd (9.6, 9.6)	71.9	4.22[b]	71.7	4.21
5	78.5	3.76 ddd (9.7, 3.8, 1.6)	78,5	3.75 m	78.1	3.93 m		3.90*
6	62.3	4.33[b]<br>4.50 brd (11.7)	62,3	4.32 brd<br>4.47 brd	63.2	4.38 dd (11.4, 5.1)<br>4.53 brd (11.4)		

Table S3 (*continued*)

	Sugar chain without OH-2 aglycone (2)		Sugar chain without OH-2 aglycone in the mix (2)		Sugar chain with OH-2 aglycone (4)		Sugar chain with OH-2 aglycone in the mix (4)	
					**Cantalasaponin-1 (1)**		**Cantalasaponin-1 (1) in mix**	
		β-D-Xyl		β-D-Xyl		β-D-Glc´		β-D-Glc´
1	105.0	5.11 d (7.7)	104.9	5.09 d (7.7)	101.7	5.09 d (7.7)	101.6	5.07 d (7.7) →77.0 (C-3Aglyc)
2	75.3	3.94 dd (8.8, 8.8)		3.91 dd (8.8, 8.8)	75.5	4.03 dd (7.7, 8.3)		4.01
3	78.6	3.99 dd (9.0, 9.0)	78.5	3.96 dd (7.9, 7.9)	78.7	4.25[b]	78.5	4.23
4	70.7	4.10[b]	70.6	4.07 m	71.8	4.23[b]		4.23
5					78.2	3.81 m	78.5	
5/6	67.3	3.63 dd (10.8; 10.8)<br>4.21 dd (11.1; 5.1)	67.2	3.61 dd (10.8; 10.8)<br>4.18 dd (11.3; 5.4)	62.7	4.30 dd (11.7, 5.0)<br>4.41 brd (11.7)		
		α-L-Rha		α-L-Rha				
1	102.8	6.10 s	102.7	6.07 brs				
2	72.4	4.66 d (3.4)		4.63 brs				
3	72.7	4.47 br d (10.9)	72.5	4.44 dd (9.3, 3.6)				
4	74.2	4.27 dd (9.6, 9.6)	74.2	4.26 dd (9.3, 9.3)				
5	69.8	4.91 dq (6.2, 9.7)	69.7	4.89 dq (9.5, 6.2)				
6	18.7	1.62 d (6.2)	18.7	1.60 d (6.1)				

[a]The assignments were confirmed by ^{1}H-^{1}H-COSY, 2D-TOCSY, HSQC, HSQC-TOCSY and HMBC experiments. [b]overlapped with other signals.

Table S4. HMAI Table of ^{13}C NMR chemical shifts for doublets. HMBC correlations with doublet signals of methyl groups C-21 and C-27. (From Simonet et al., *Phytochem. Anal.*, 2021: *32*, 38-61).

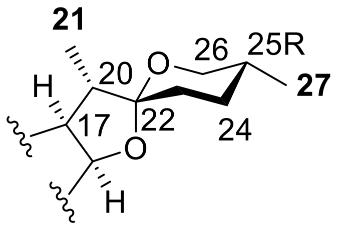

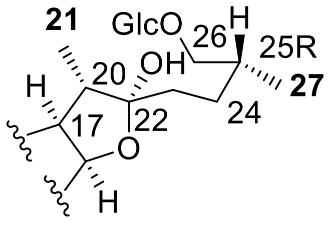

Structural Features						HMBC signals								
C-9	C-12	C-23	C-24	C-22	C-25	**H-27**	C-24	C-25	C-26	**H-21**	C-17	C-20	C-22	Data
				SP	R	0.67	29.1	30.6	66.7	1.12	62.9	41.9	109.3	**1**
				SP	DB	-	-	-	-	1.08	63.2	41.9	109.4	**11**
				F	R	0.96	28.5	34.4	75.3	1.30	63.9	40.8	110.7	**43**
				FM	R	0.98	28.2	34.2	75.2	1.16	64.1	40.5	112.7	**44**
		OHα		SP	R	0.72	38.9	31.8	66.0	1.16	62.6	35.9	111.7	**58**
			OGlc*β*	SP	R*	1.12	81.5	38.2	65.1	1.02	62.3	42.1	111.6	**62**
		OHα	OGlc*β*	SP	R*	1.19	87.9	37.9	64.1	1.15	62.0	34.6	112.7	**60**
	CO			SP	S	1.05	26.2	27.5	65.2	1.35	54.2	43.1	109.8	**24**
	CO			SP	R	0.64	29.2	30.5	66.9	1.31	54.3	42.6	109.3	**18**
	CO			F	R	0.96	28.4	34.3	75.3	1.53	54.9	41.3	110.9	**32**
	CO			F	S	1.01	28.3	34.4	75.3	1.51	54.8	41.3	110.8	**34**
DB	CO			SP	R	0.67	29.2	30.5	67.0	1.38	54.5	43.0	109.5	**28**
	OH*β*			SP	R	0.67	29.4	30.7	66.9	1.41	63.0	43.1	109.6	**47**

OH: hydroxyl; DB: double bond; CO: carbonyl; SP: spirostanic; F: furostanic; R/S/*α*/*β*: chiral center configuration.

* R is the relative configuration; S is the absolute configuration because a glucopyranosyloxy moiety is at C-24.

Table S5. HMAI Table of ^{13}C NMR chemical shifts for singlets. HMBC correlations with singlet signals of methyl groups C-18 and C-19. (From Simonet et al., *Phytochem. Anal.*, 2021: *32*, 38-61).

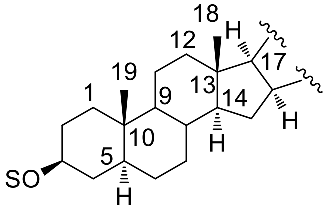

Structural Features								HMBC signals										
C-2	C-5	C-6	C-9	C-12	C-22	C-23	C-24	**H-18**	C-12	C-13	C-14	C-17	**H-19**	C-1	C-5	C-9	C-10	Data
	α				SP			0.80	40.0	40.5	56.1	62.9	0.62	36.9	44.4	54.1	35.6	**1**
	α				SP	OH*α*	OGlc*β*	1.01	40.7	41.4	56.6	62	0.75	37.5	45.6	54.6	35.9	**60**
	α			CO	SP			1.03	212.8	55.4	55.9	54.3	0.61	36.6	44.4	55.5	36.3	**18**
	α		DB	CO	SP			0.98	204.3	51.3	52.7	54.5	0.79	35.0	42.5	171.3	39.5	**28**
OH *α*	α		DB	CO	SP			0.97	204.3	51.4	52.7	54.6	0.86	43.5	42.5	170.5	40.6	**55**
	α			OH*β*	SP			1.06	79.3	46.6	55.2	63.0	0.64	37.2	44.7	53.6	35.9	**47**
OH *α*	α				SP			0.78	40.0	40.6	56.3	63.0	0.69	45.6	44.6	54.3	36.8	**35**
	β				SP			0.79	40.3	40.9	56.5	63.1	0.84	30.8	36.9	40.2	35.2	**4**
OH *β*	β				SP			0.77	40.2	40.8	56.3	63.1	0.87	40.5	36.4	41.4	36.9	**38**
	DB				SP			0.80	39.9	40.5	56.7	62.9	0.85	37.5	141.1	50.3	37.1	**9**
	DB			CO	F			1.13	212.9	55.4	56.0	54.9	0.91	37.0	140.9	52.4	37.6	**32**
OH *α*	DB				SP			0.78	39.8	40.5	56.5	62.9	0.91	45.8	140.1	50.2	38.0	**40**
OH *α*	DB				SP		OGlc*β*	0.71	39.7	40.4	56.5	62.3	0.91	45.7	140.1	50.1	37.9	**62**
OH *α*	DB				F			0.85	39.9	40.8	56.5	63.9	0.92	45.8	140.1	50.3	38.0	**43**
OH *α*	DB				FM			0.77	39.6	40.8	56.4	64.1	0.91	45.7	140.1	50.2	37.9	**44**
	α	OH*α*			SP			0.81	40.0	40.7	56.2	62.9	0.68	37.6	52.1	54.0	36.4	**45**
	α	OGlc*α*			SP			0.76	40.0	40.8	56.4	63.0	0.68	37.5	50.9	53.8	36.7	**46**
	α	OGlc*α*			SP	OH*α*		0.96	22.7	41.4	56.5	62.6	0.74	37.8	51.3	54.0	36.8	**58**

OH: hydroxyl; DB: double bond; CO: carbonyl; SP: spirostanic; F: furostanic; *α*/*β*: chiral center configuration.

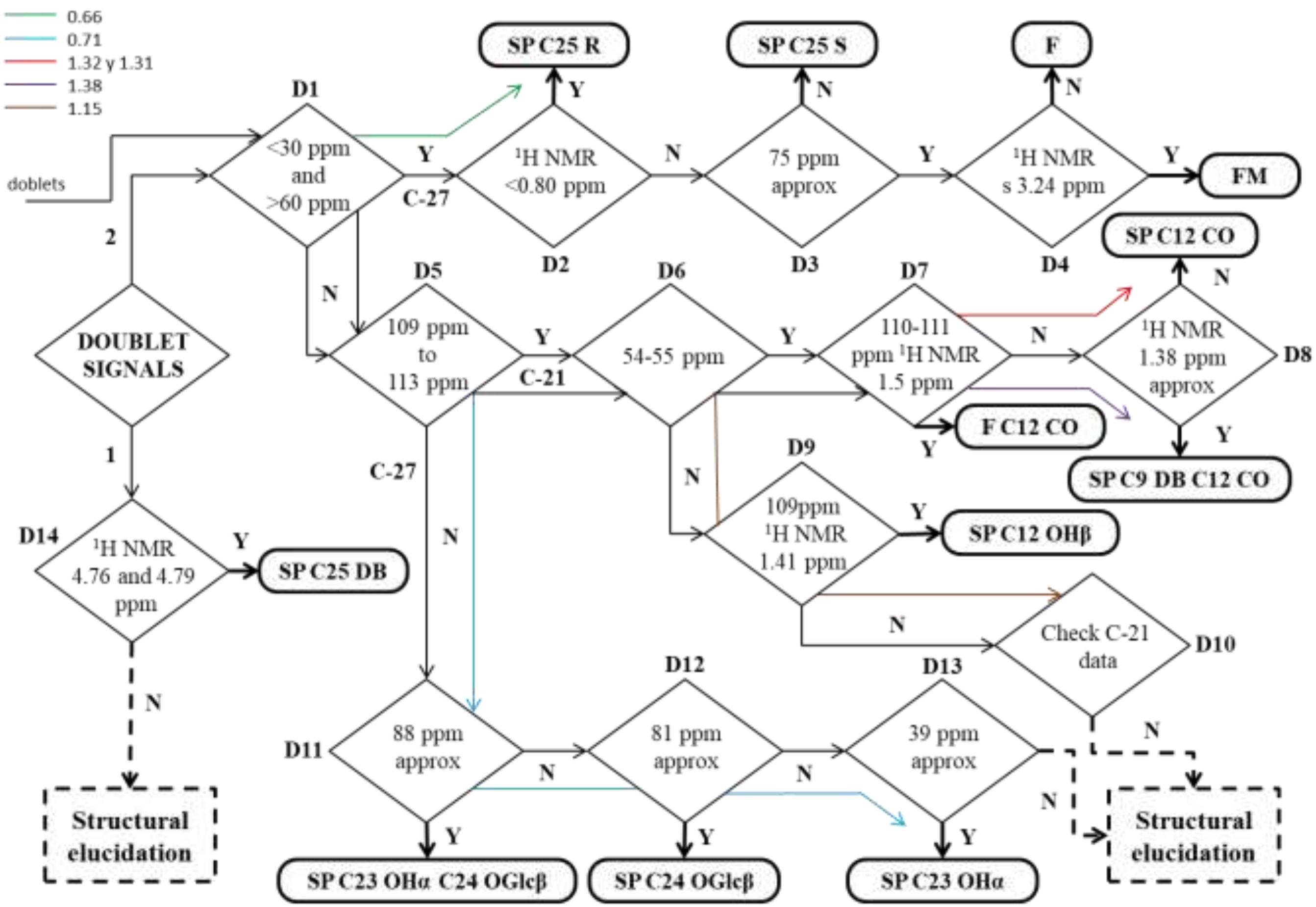

SP: spirostanic; F furostanic; FM: methoxyfurostanic; R/S/α/β quiral configuration; CO: carbonyl group; OH: hydroxyl group; DB: double bound; Oglc: glucopyranosyloxy; Y: Yes; N: Not.

Figure S1. HMAI Flowchart of doublets with notes.

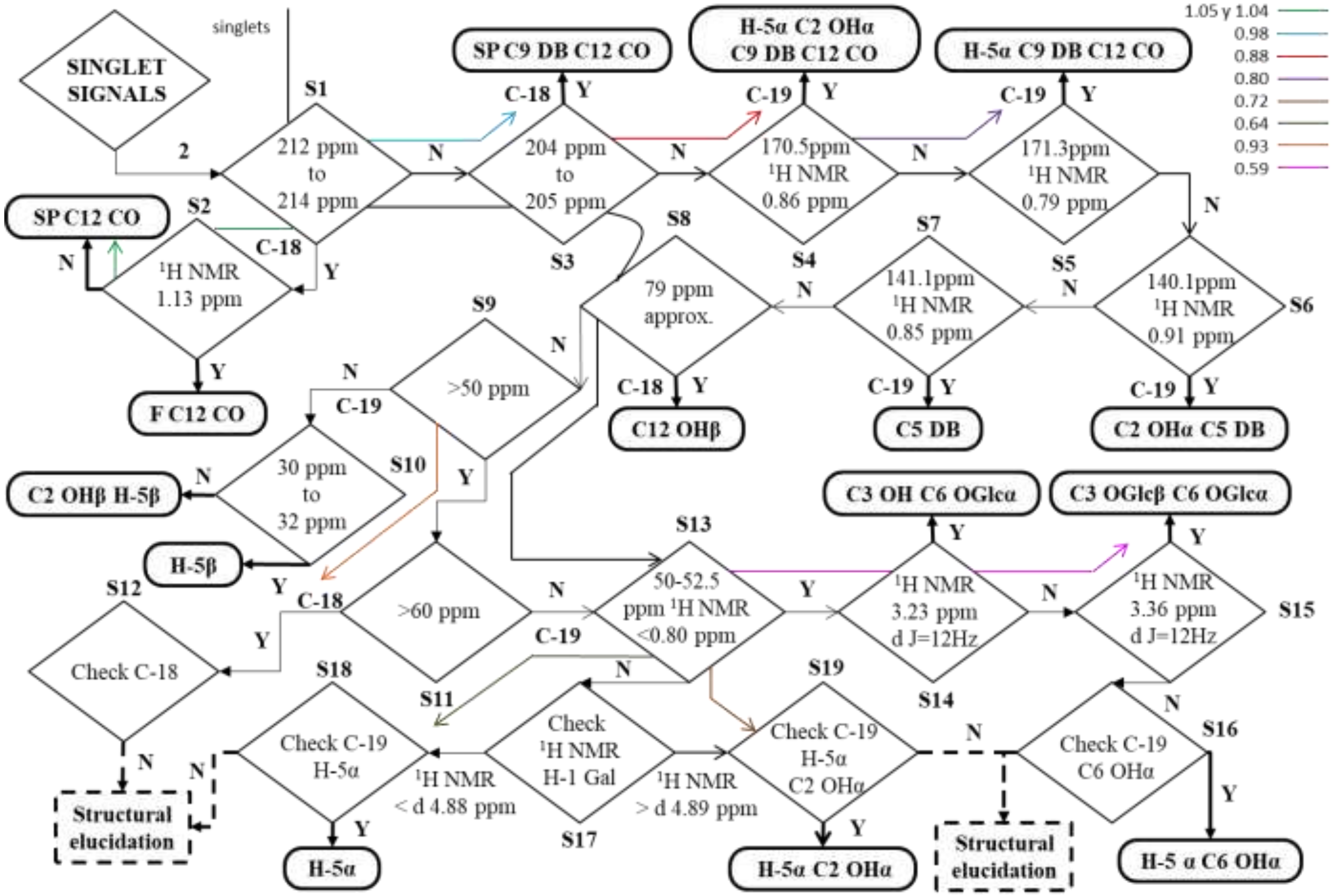

SP: spirostanic; F furostanic; R/S/α/β quiral configuration; CO: carbonyl group; OH: hydroxyl group; DB: double bound; Oglc: glucopyranosyloxy; Y: Yes; N: Not.

Figure S2. HMAI Flowchart of singlets with notes.

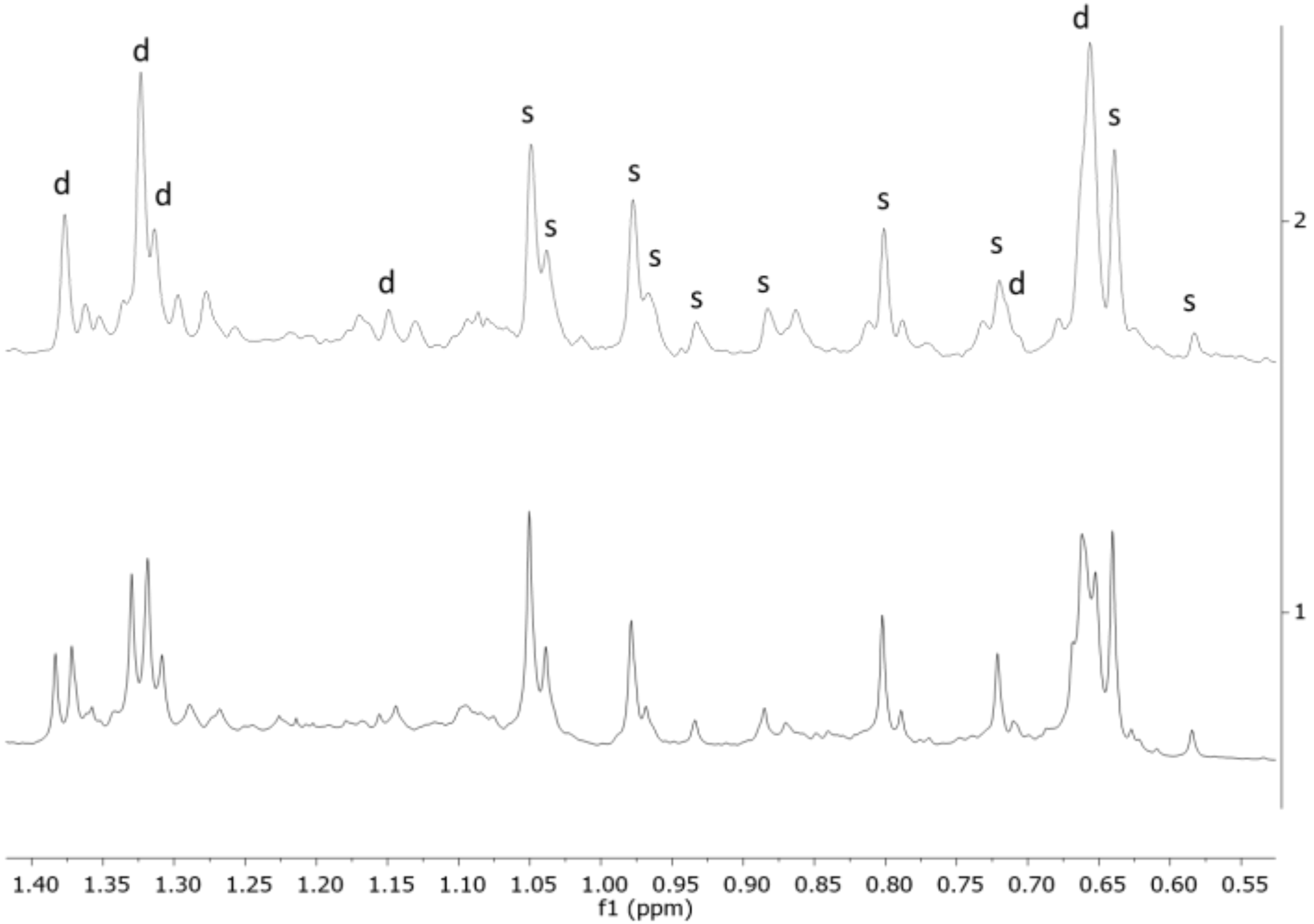

Figure S3. ^{1}H NMR and Pure shift 1D NMR spectra of saponin-enriched fraction of *A. macroacantha* (0.55 to 1.40 ppm; 600 MHz, Pyridine-d_5).

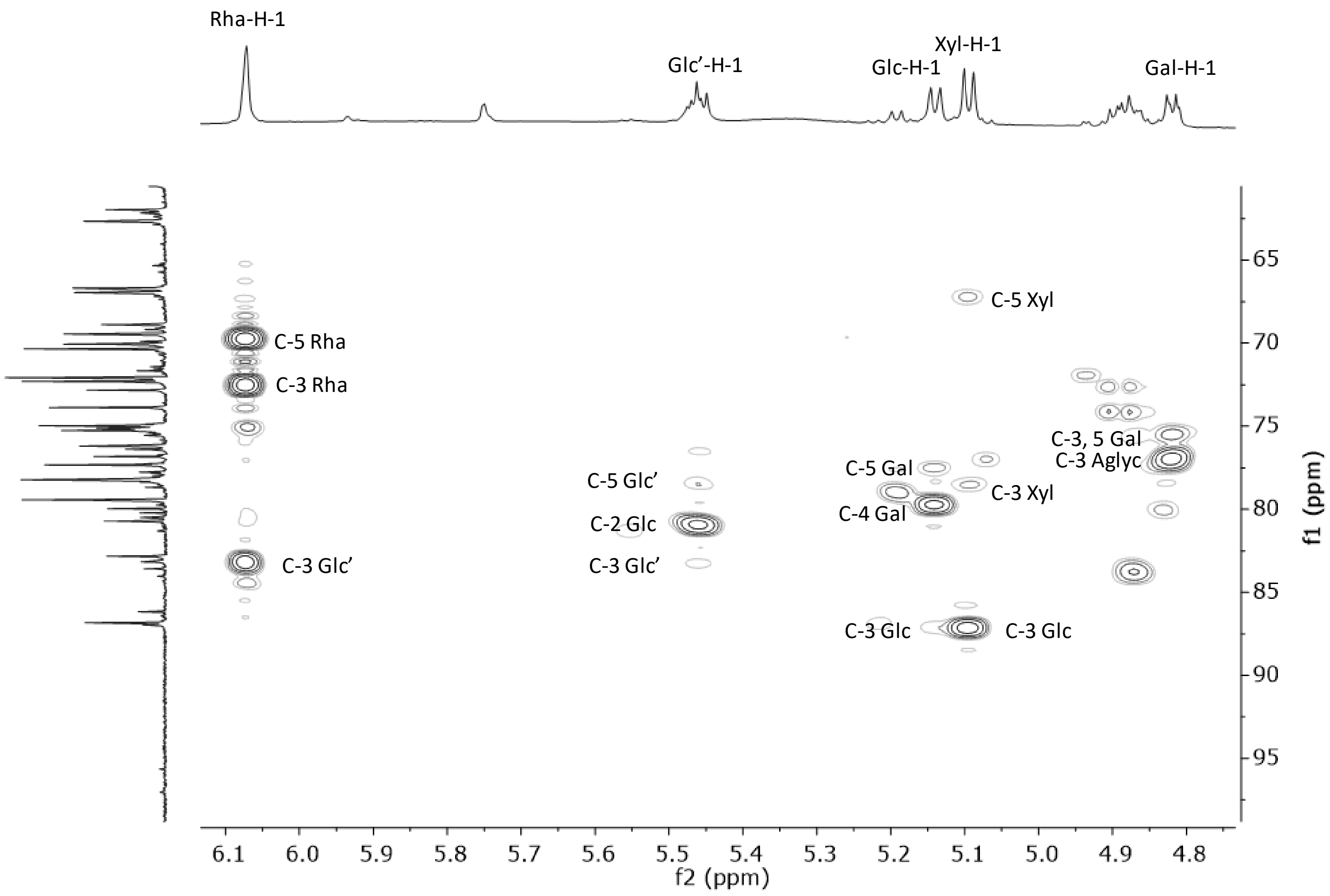

Figure S4. HMBC of saponin-enriched fraction of *Agave macroacantha* for sugar residues (70 to 100 ppm; 600 MHz, Pyridine-d_5).

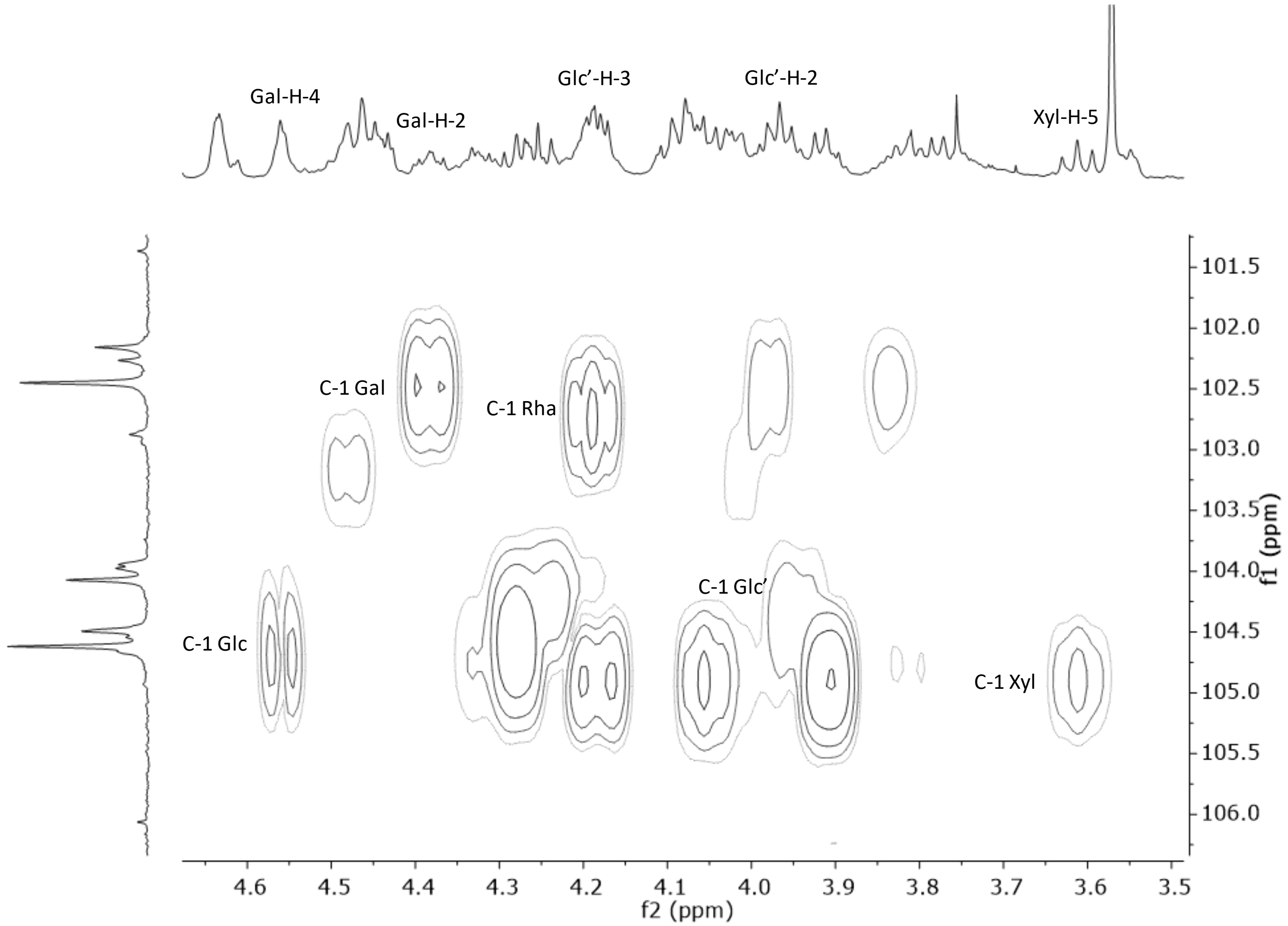

Figure S5. HMBC of saponin-enriched fraction of *Agave macroacantha* for sugar residues (102.0 to 106.5 ppm; 600 MHz, Pyridine-d_5).

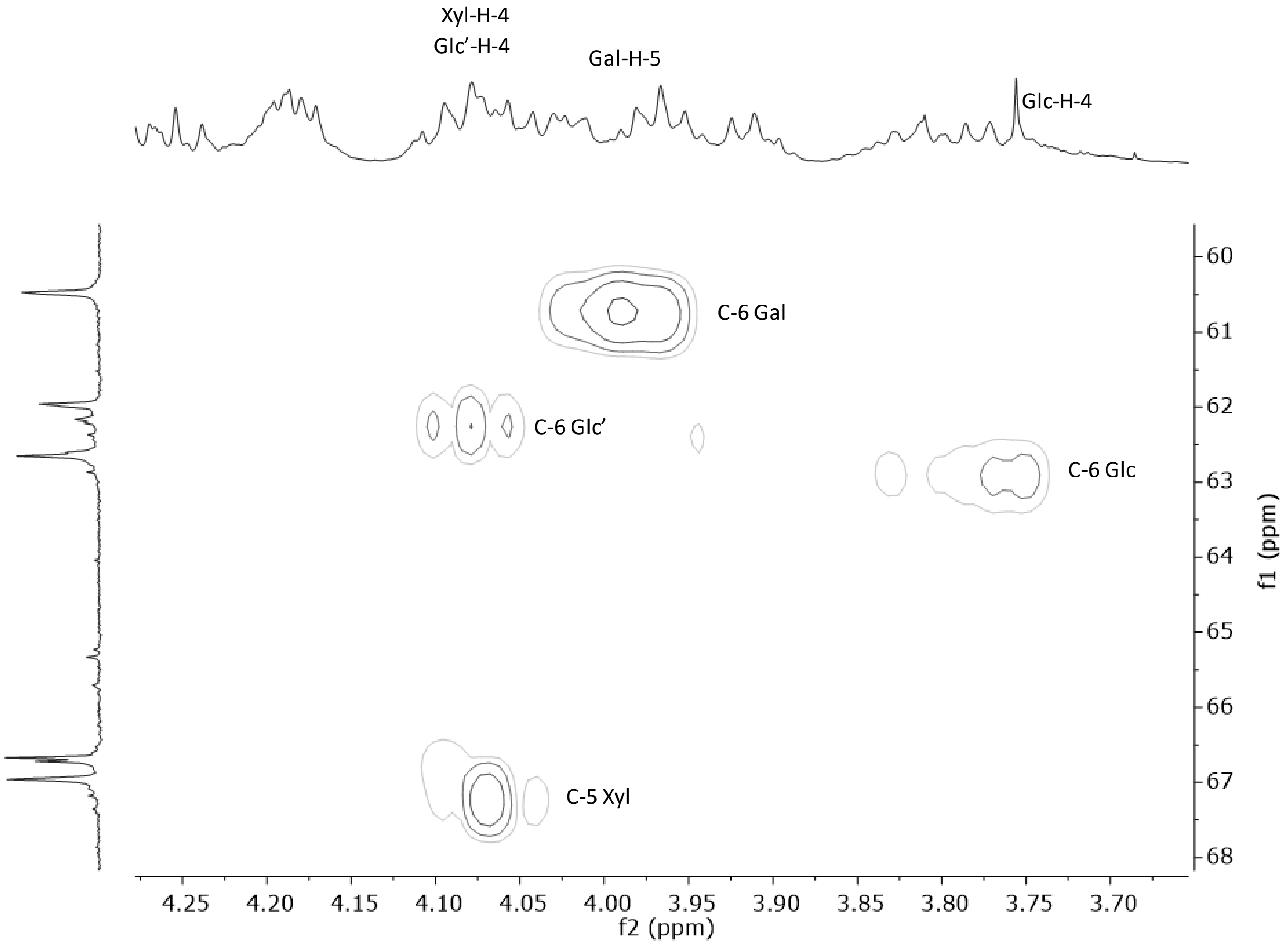

Figure S6. HMBC of saponin-enriched fraction of *Agave macroacantha* for sugar residues (59.5 to 68 ppm; 600 MHz, Pyridine-d_5).

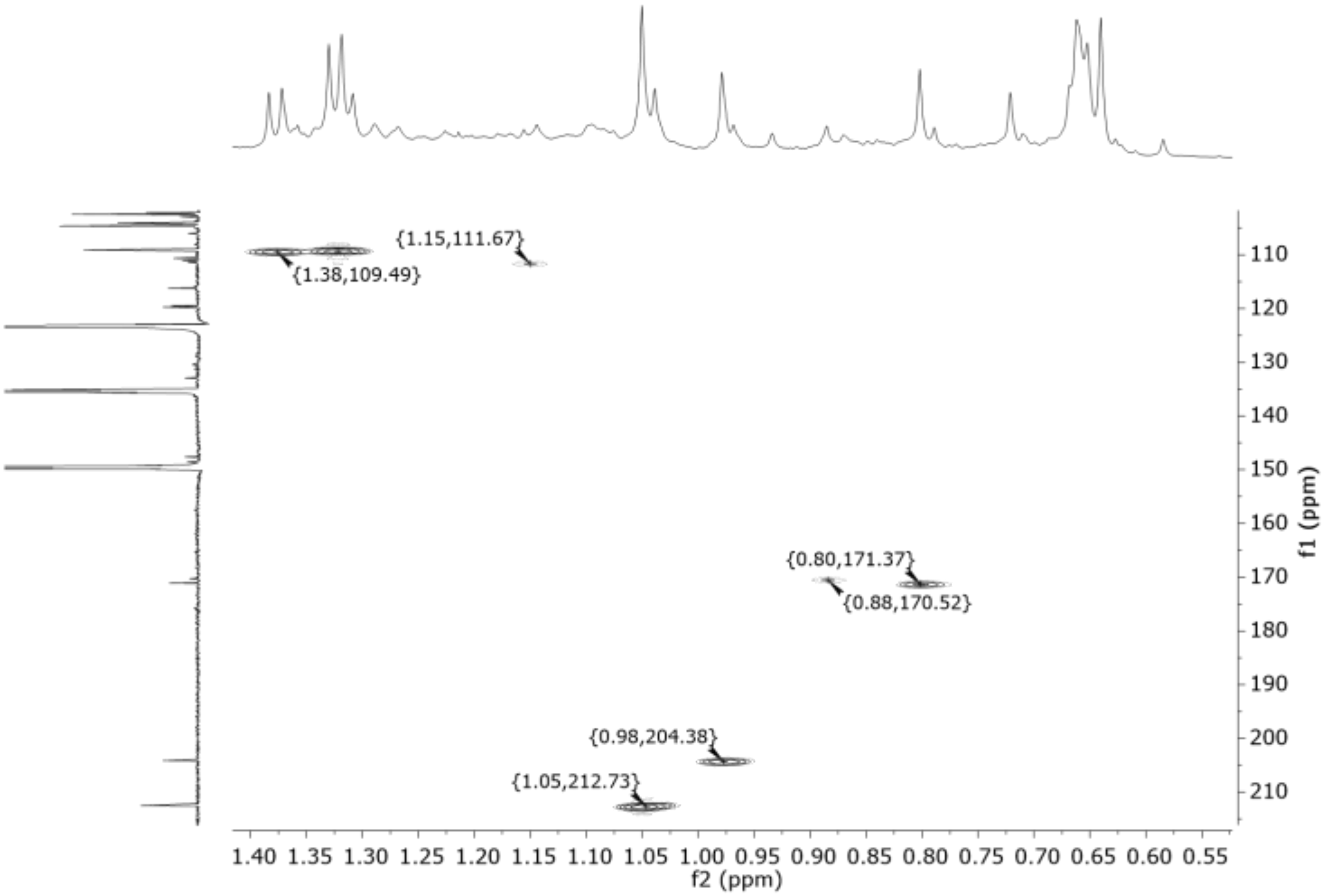

Figure S7. HMBC of saponin-enriched fraction of *Agave macroacantha* (215 to 100 ppm; 600 MHz, Pyridine-d_5).

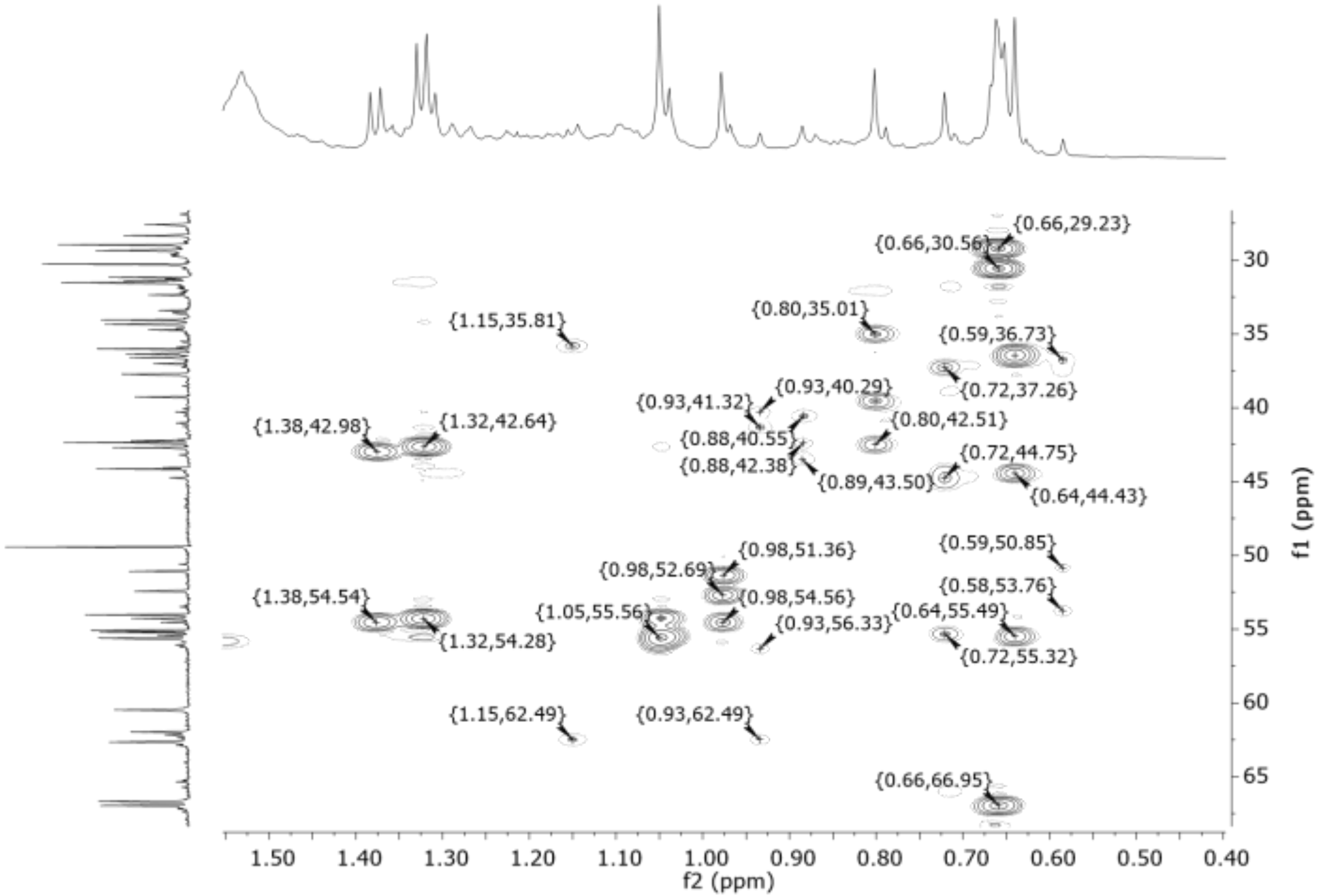

Figure S8. HMBC of saponin-enriched fraction of *A. macroacantha* (70 to 25 ppm; 600 MHz, Pyridine-d_5).

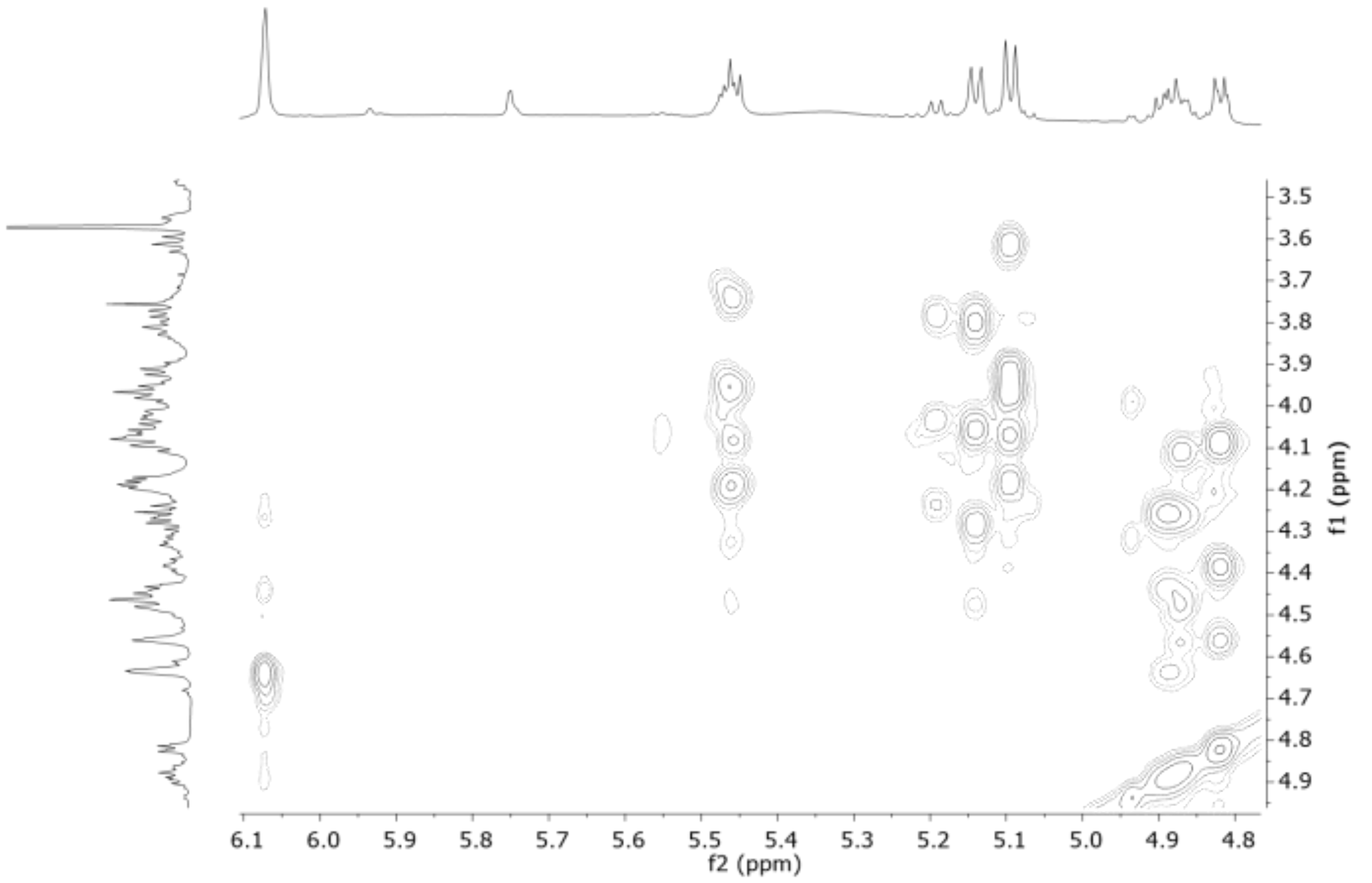

Figure S9. 2D TOCSY spectrum of saponin-enriched fraction of *A. macroacantha* (4.80 to 6.10 ppm; 600 MHz, Pyridine-d_5).

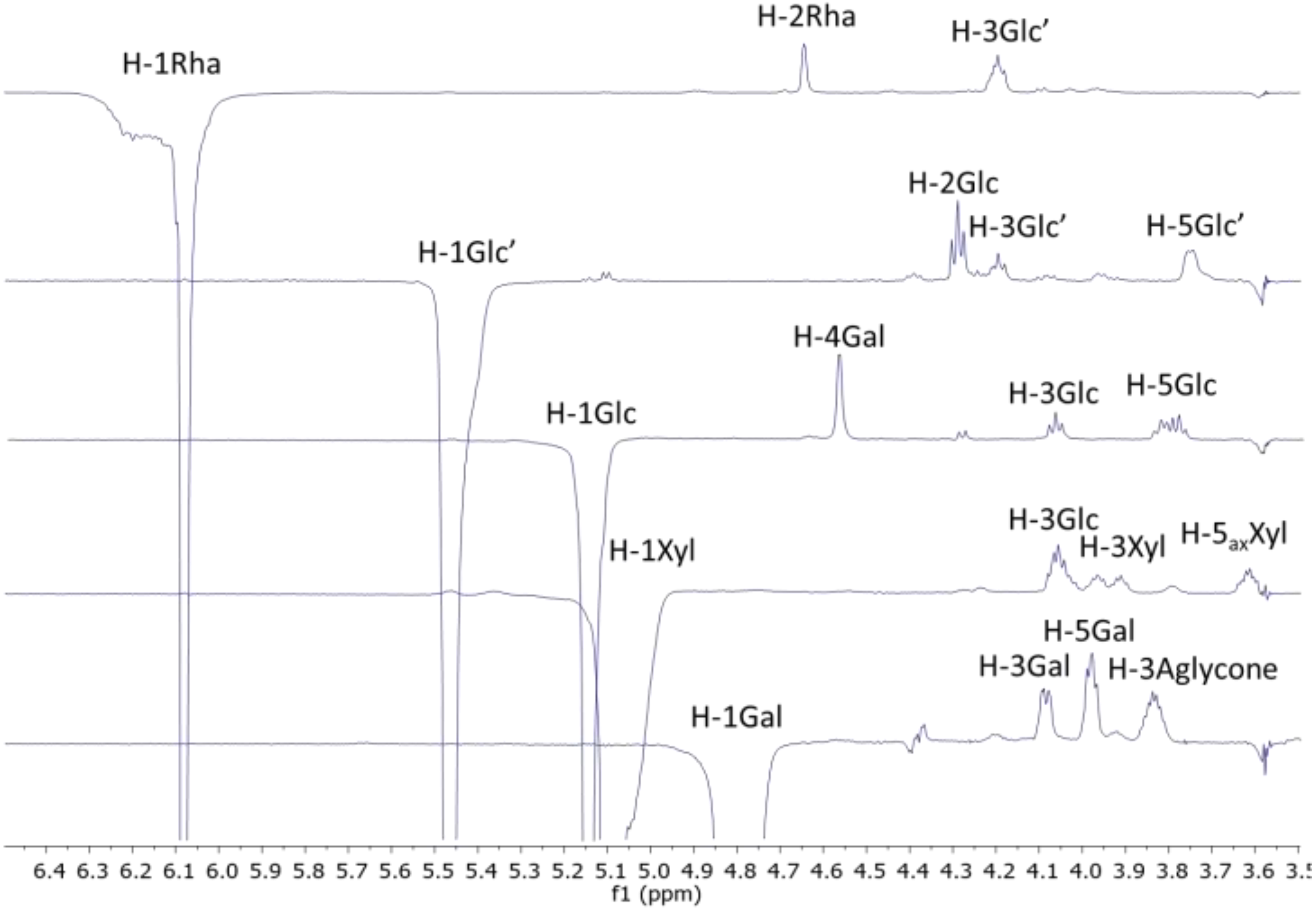

Figure S10. Selected 1D ROESY spectra of saponin-enriched fraction of *A. macroacantha* (600 MHz, Pyridine-d_5).

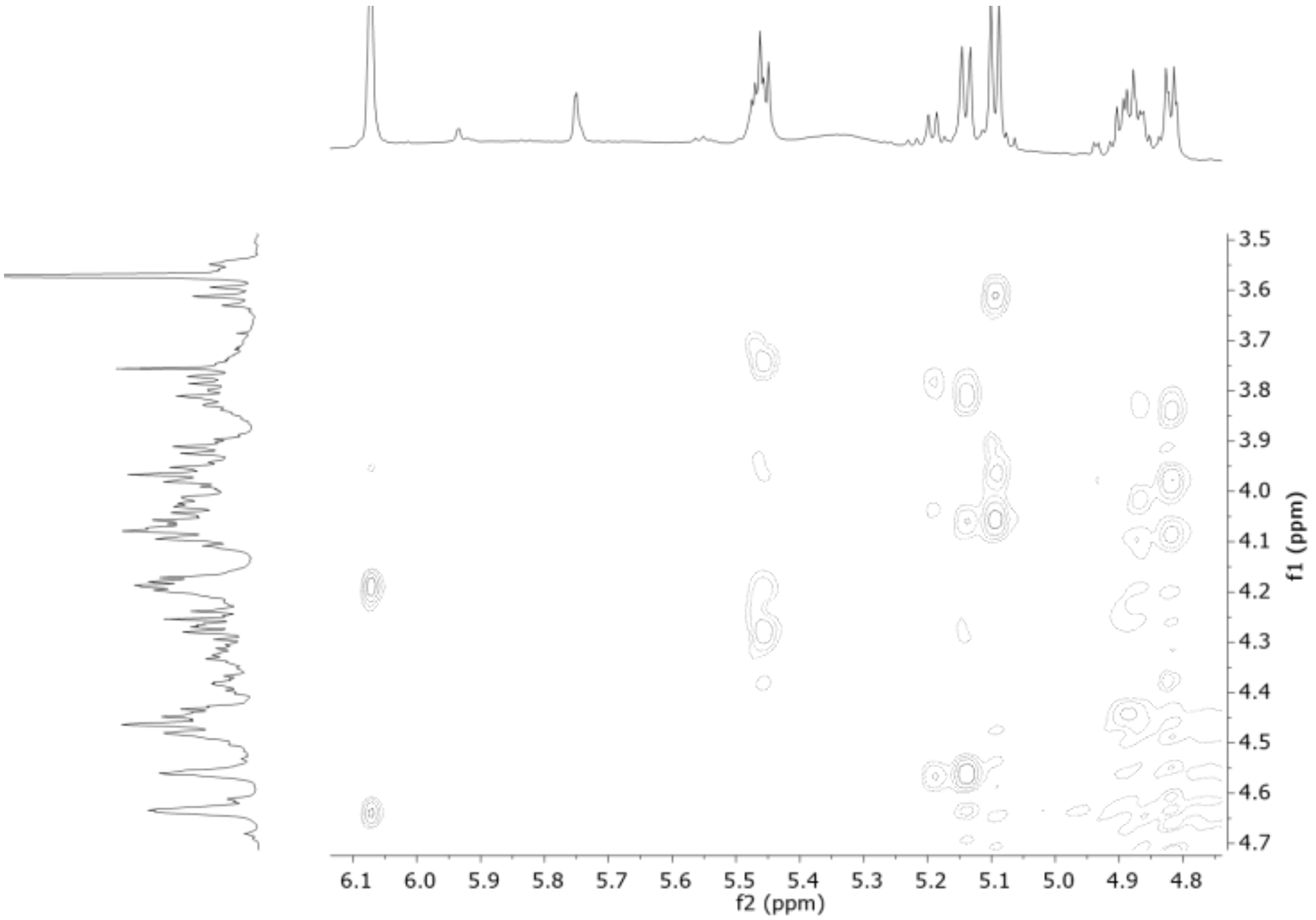

Figure S11. 2D ROESY spectrum of saponin-enriched fraction of *A. macroacantha* (4.80 to 6.10 ppm; 600 MHz, Pyridine-d_5).

Elemental Composition Report

Page 1

Single Mass Analysis

Tolerance = 5.0 mDa / DBE: min = -1.5, max = 50.0
Element prediction: Off
Number of isotope peaks used for i-FIT = 3

Monoisotopic Mass, Even Electron Ions
618 formula(e) evaluated with 3 results within limits (up to 50 best isotopic matches for each mass)
Elements Used:
C: 0-1000 H: 0-1000 O: 0-100
26-ENERO-2021
M14 160 (2.967)

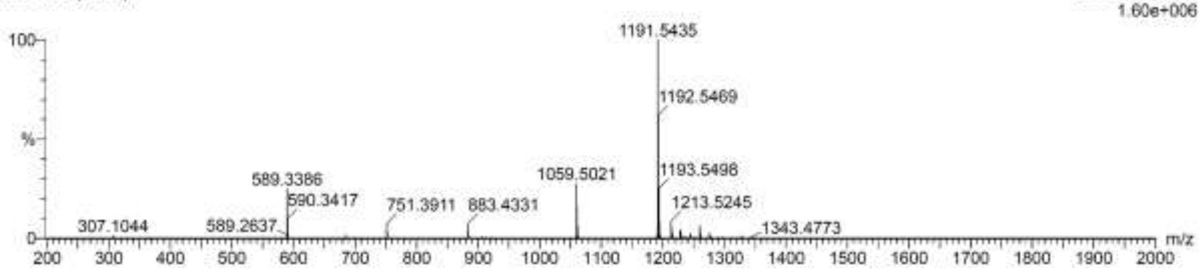

Minimum:				-1.5					
Maximum:		5.0	10.0	50.0					
Mass	Calc. Mass	mDa	PPM	DBE	i-FIT	Norm	Conf(%)	Formula	
1191.5435	1191.5435	0.0	0.0	13.5	387.8	0.000	100.00	C56 H87 O27	
	1191.5470	-3.5	-2.9	35.5	402.3	14.531	0.00	C74 H79 O14	
	1191.5411	2.4	2.0	44.5	402.4	14.558	0.00	C81 H75 O9	

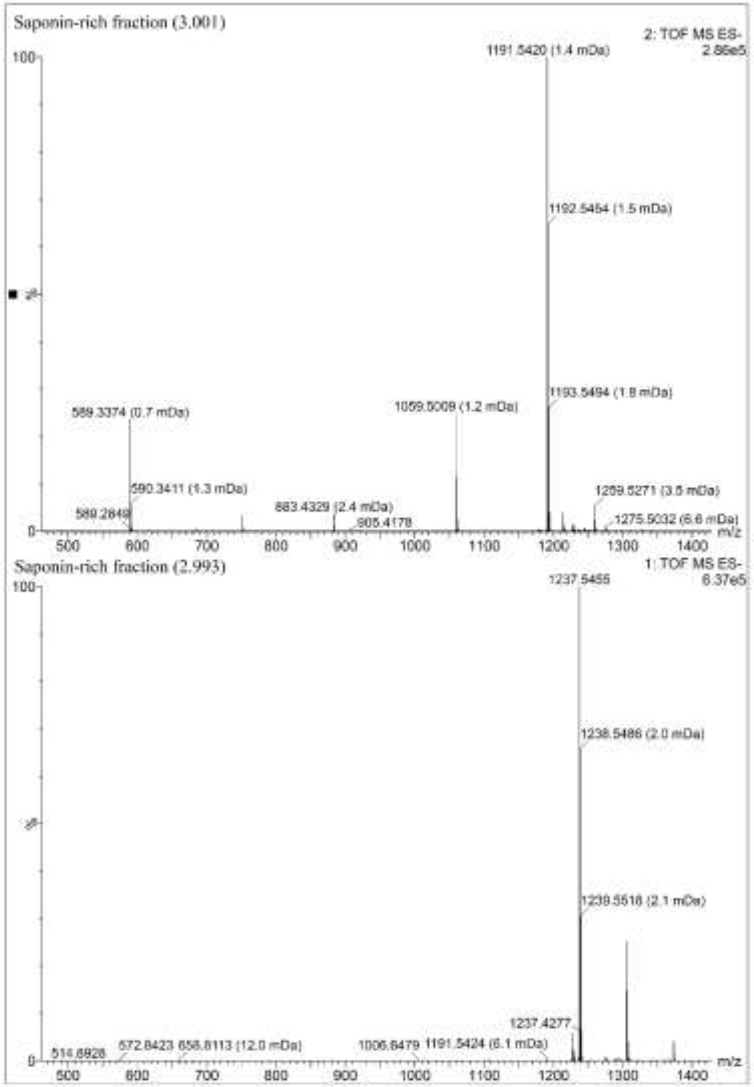

Figure S12. Chromatogram and HRESI MS (negative mode) of Macroacanthoside A (**3**) calculated for $C_{56}H_{87}O_{27}$ [M – H]$^-$: 1191.5435, found 1191.5435.

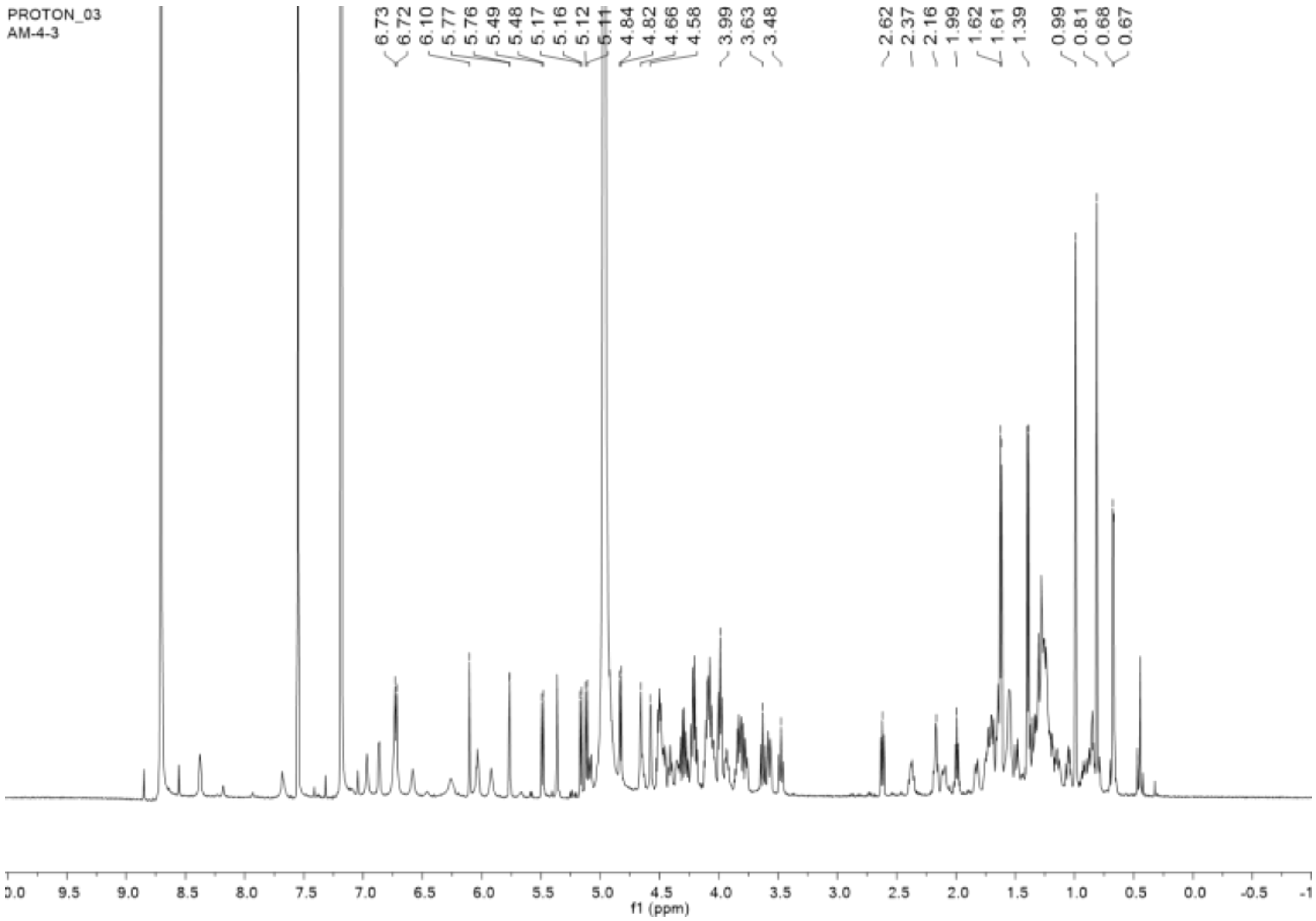

Figure S13. ^{1}H NMR spectrum of Macroacanthoside A (**3**) (600 MHz, Pyridine-d_5).

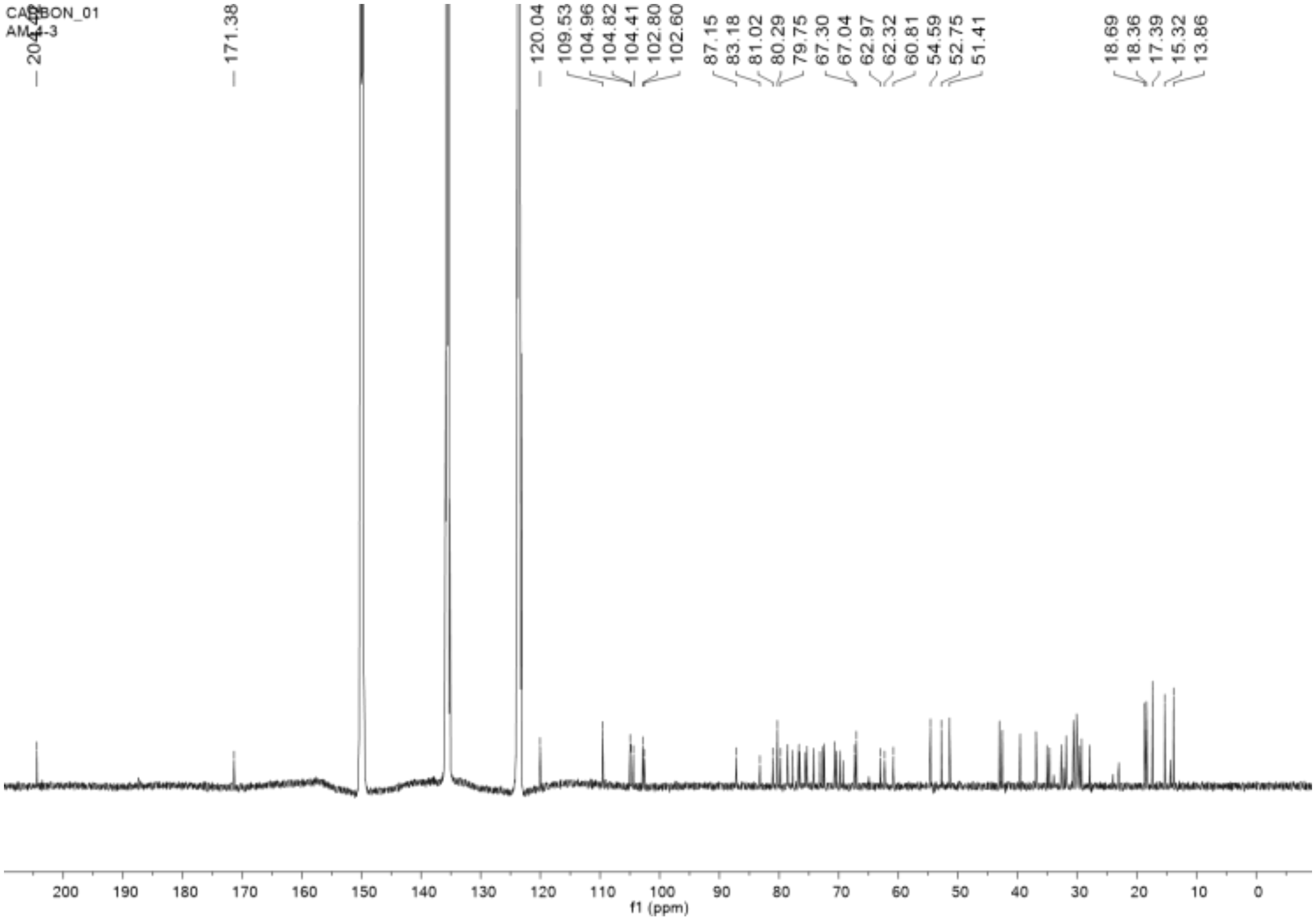

Figure S14. ^{13}C NMR spectrum of Macroacanthoside A (**3**) (600 MHz, Pyridine-d_5).

Elemental Composition Report **Page 1**

Single Mass Analysis
Tolerance = 5.0 mDa / DBE: min = -1.5, max = 50.0
Element prediction: Off
Number of isotope peaks used for i-FIT = 3

Monoisotopic Mass, Even Electron Ions
622 formula(e) evaluated with 3 results within limits (up to 50 best isotopic matches for each mass)
Elements Used:
C: 0-1000 H: 0-1000 O: 0-100
26-ENERO-2021
M14 126 (2.338)

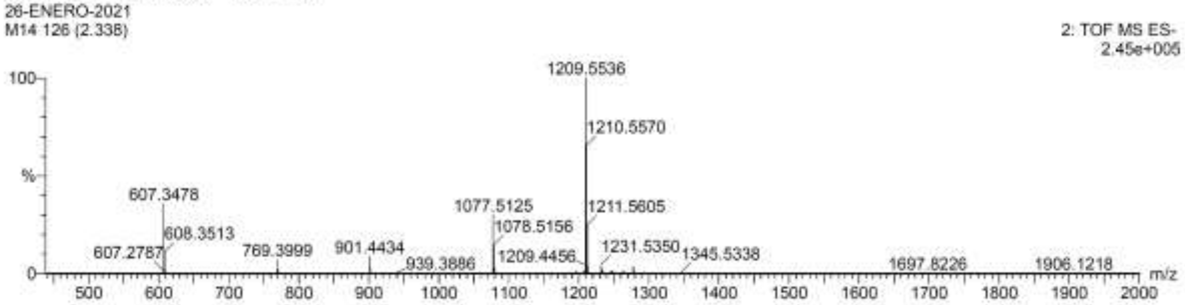

Minimum:				-1.5				
Maximum:		5.0	10.0	50.0				
Mass	Calc. Mass	mDa	PPM	DBE	i-FIT	Norm	Conf(%)	Formula
1209.5536	1209.5540	-0.4	-0.3	12.5	396.3	0.000	99.99	C56 H89 O28
	1209.5517	1.9	1.6	43.5	405.7	9.401	0.01	C81 H77 O10
	1209.5575	-3.9	-3.2	34.5	405.9	9.609	0.01	C74 H81 O15

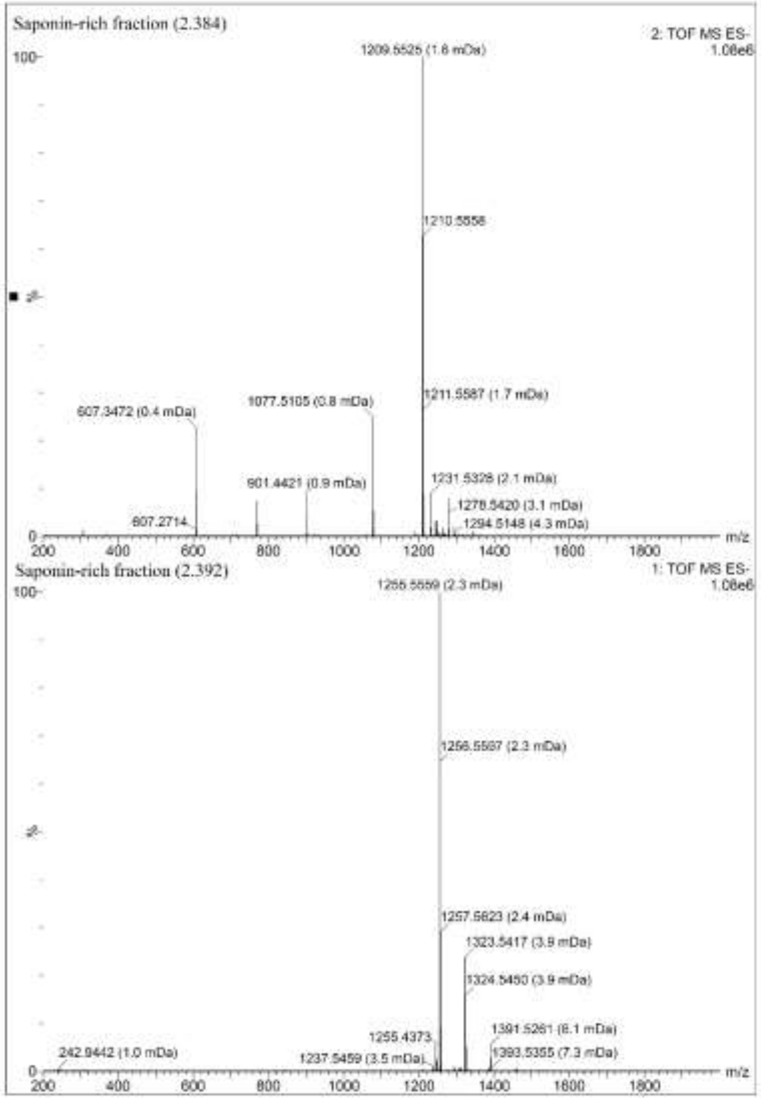

Figure S15. Chromatogram and HRESI MS (negative mode) of Macroacanthoside B (**4**) calculated for $C_{56}H_{89}O_{28}$ [M – H]⁻: 1209.5540, found 1209.5536.

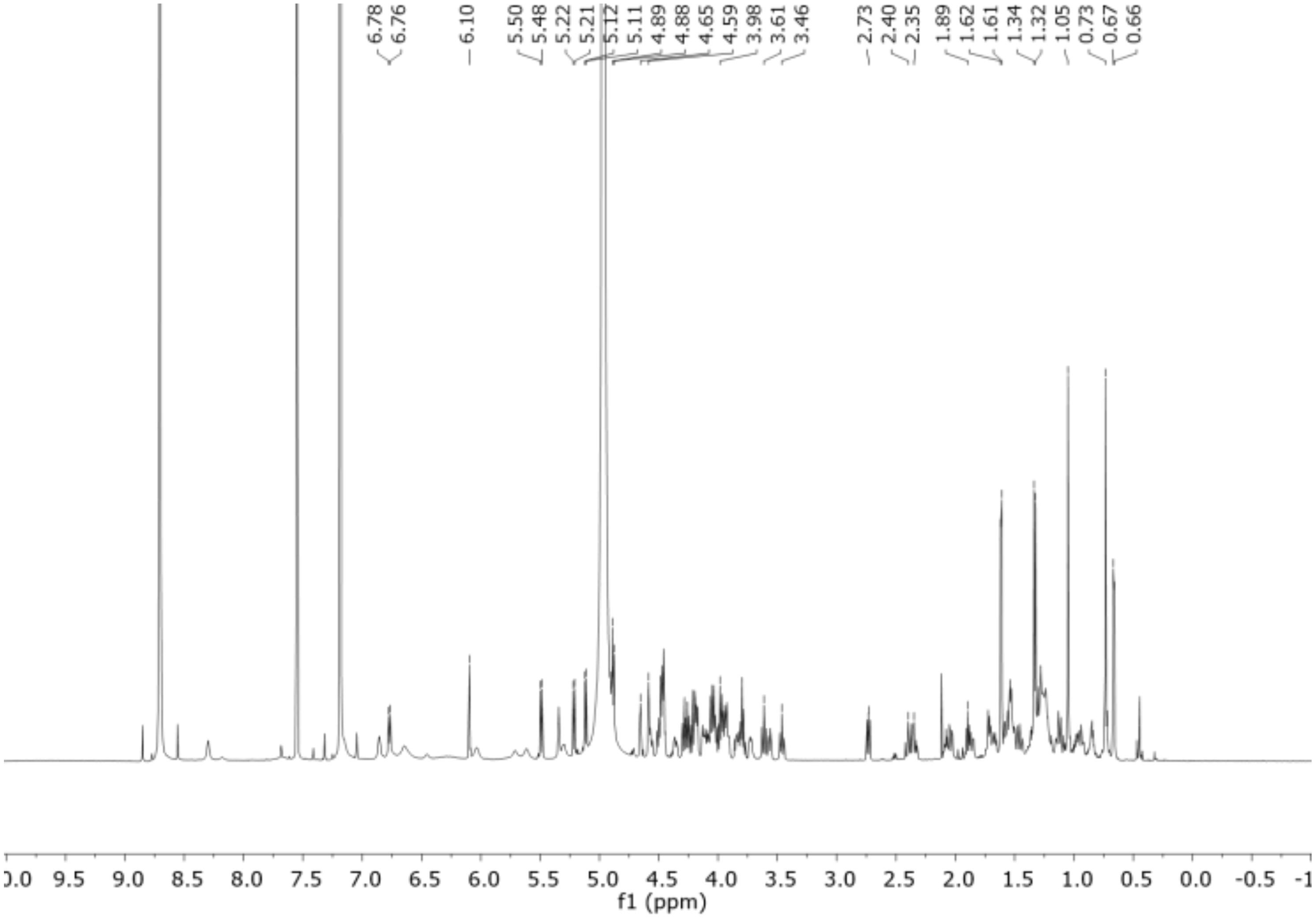

Figure S16. ^{1}H NMR spectrum of Macroacanthoside B (**4**) (600 MHz, Pyridine-d_5).

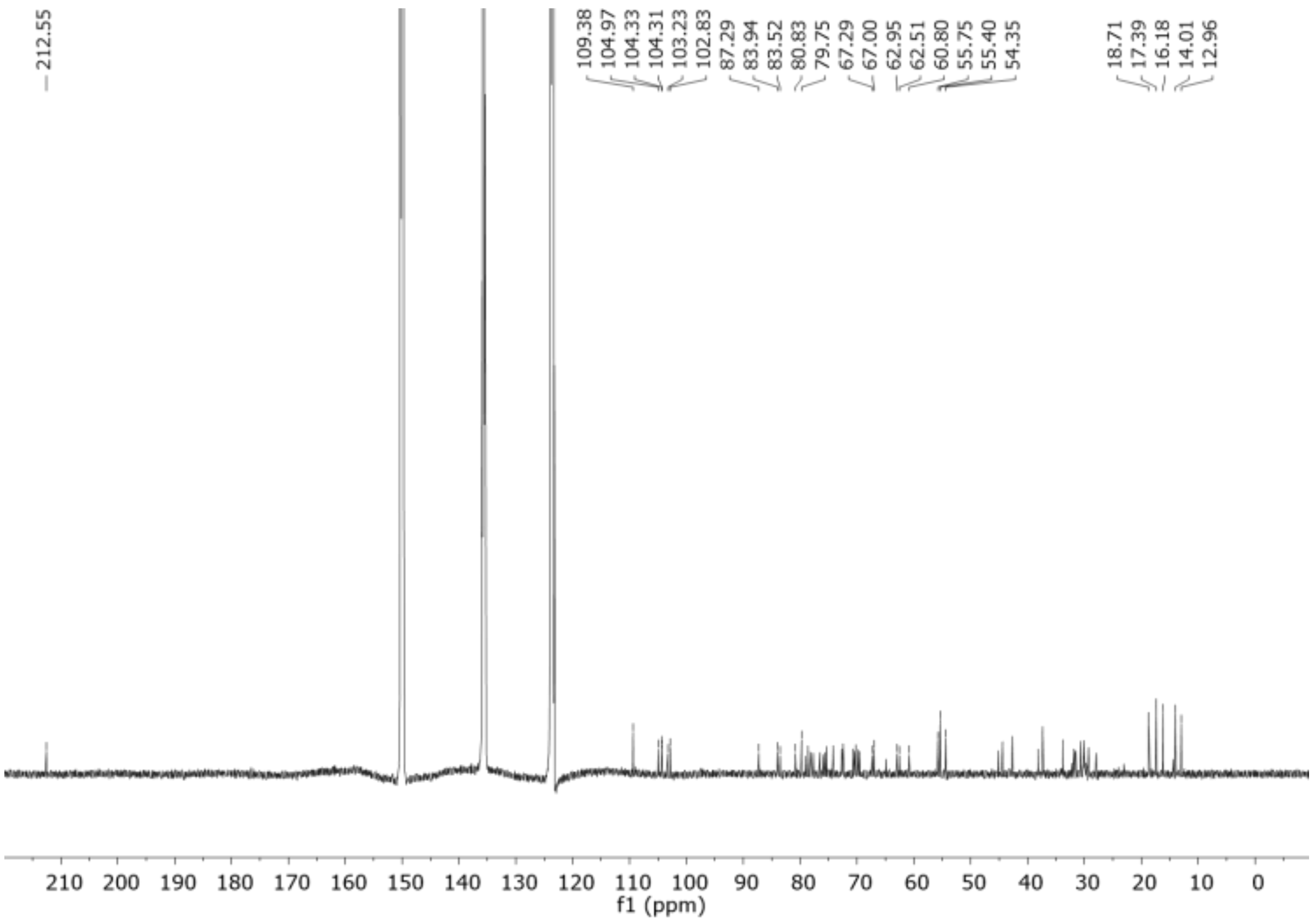

Figure S17. ^{13}C NMR spectrum of Macroacanthoside B (**4**) (600 MHz, Pyridine-d_5).

Elemental Composition Report **Page 1**

Single Mass Analysis
Tolerance = 5.0 mDa / DBE: min = -1.5, max = 50.0
Element prediction: Off
Number of isotope peaks used for i-FIT = 3

Monoisotopic Mass, Even Electron Ions
633 formula(e) evaluated with 4 results within limits (up to 50 best isotopic matches for each mass)
Elements Used:
C: 0-1000 H: 0-1000 O: 0-100
26-ENERO-2021
M14 112 (2.086)

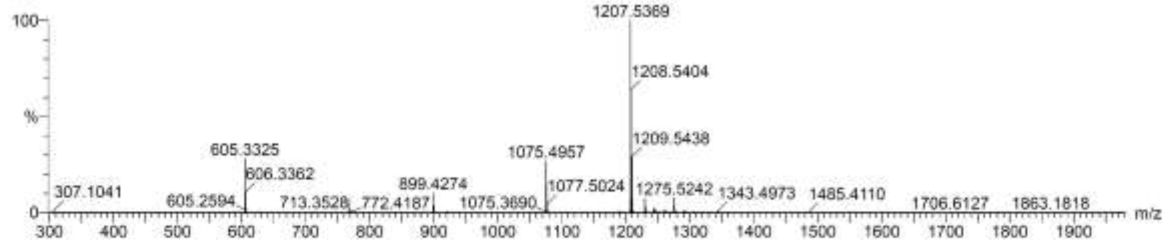

Minimum:				-1.5				
Maximum:		5.0	10.0	50.0				
Mass	Calc. Mass	mDa	PPM	DBE	i-FIT	Norm	Conf(%)	Formula
1207.5369	1207.5384	-1.5	-1.2	13.5	446.5	0.109	89.67	C56 H87 O28
	1207.5325	4.4	3.6	22.5	448.8	2.359	9.45	C63 H83 O23
	1207.5360	0.9	0.7	44.5	451.3	4.928	0.72	C81 H75 O10
	1207.5419	-5.0	-4.1	35.5	452.9	6.461	0.16	C74 H79 O15

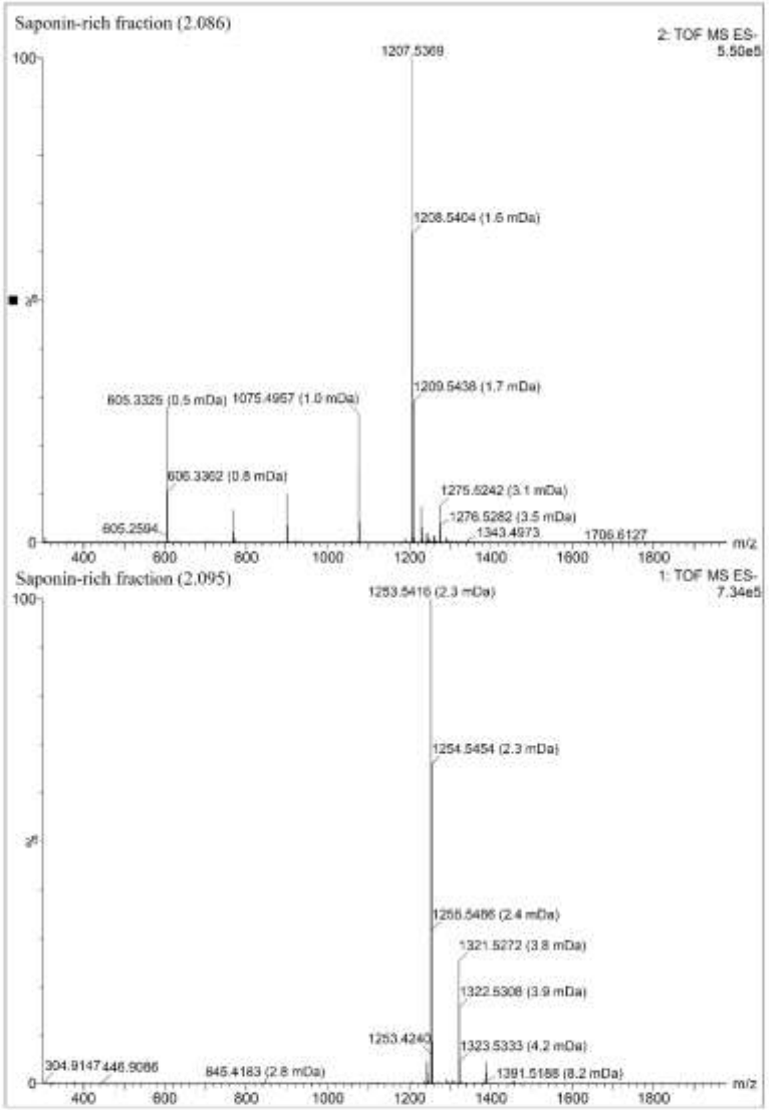

Figure S18. Chromatogram and HRESI MS (negative mode) of Macroacanthoside C (**5**) calculated for $C_{56}H_{87}O_{28}$ [M – H]⁻: 1207.5384, found 1207.5369.

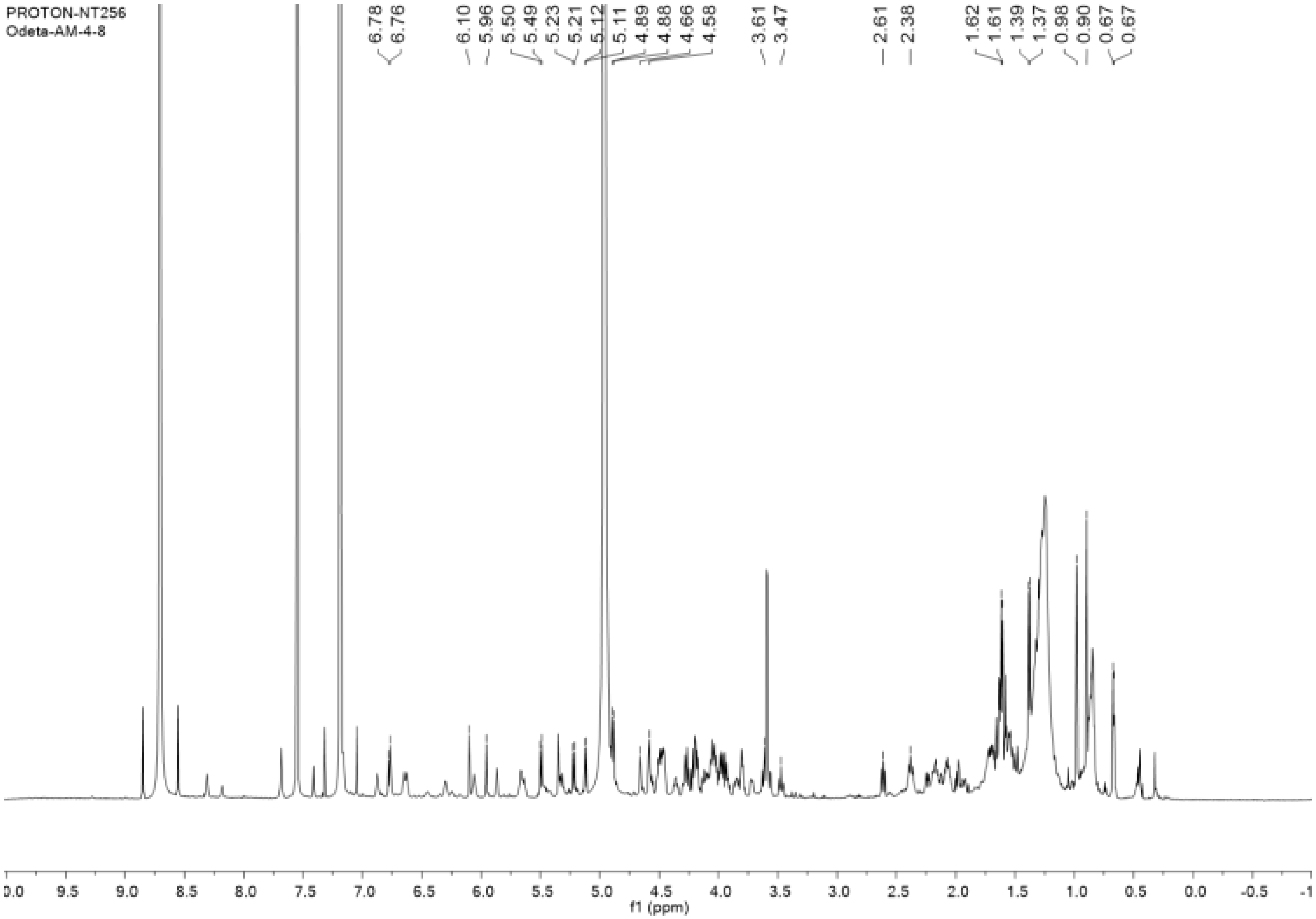

Figure S19. ^{1}H NMR spectrum of Macroacanthoside C (**5**) (600 MHz, Pyridine-d_5).
